# Developing an *E. coli*-Based Cell-Free Protein Synthesis System for Artificial Spidroin Production and Characterization

**DOI:** 10.1021/acssynbio.5c00241

**Published:** 2025-04-21

**Authors:** Chang-Yen Huang, Ruei-Chi Wang, Tzy-Shyuan Hsu, Tzu-Ning Hung, Ming-Yan Shen, Chung-Heng Chang, Hsuan-Chen Wu

**Affiliations:** Department of Biochemical Science and Technology, National Taiwan University, No. 1, Section 4, Roosevelt Road, Taipei 10617, Taiwan (ROC)

**Keywords:** cell-free protein synthesis, spidroin, spider silk, self-assembly, phase separation, fiber

## Abstract

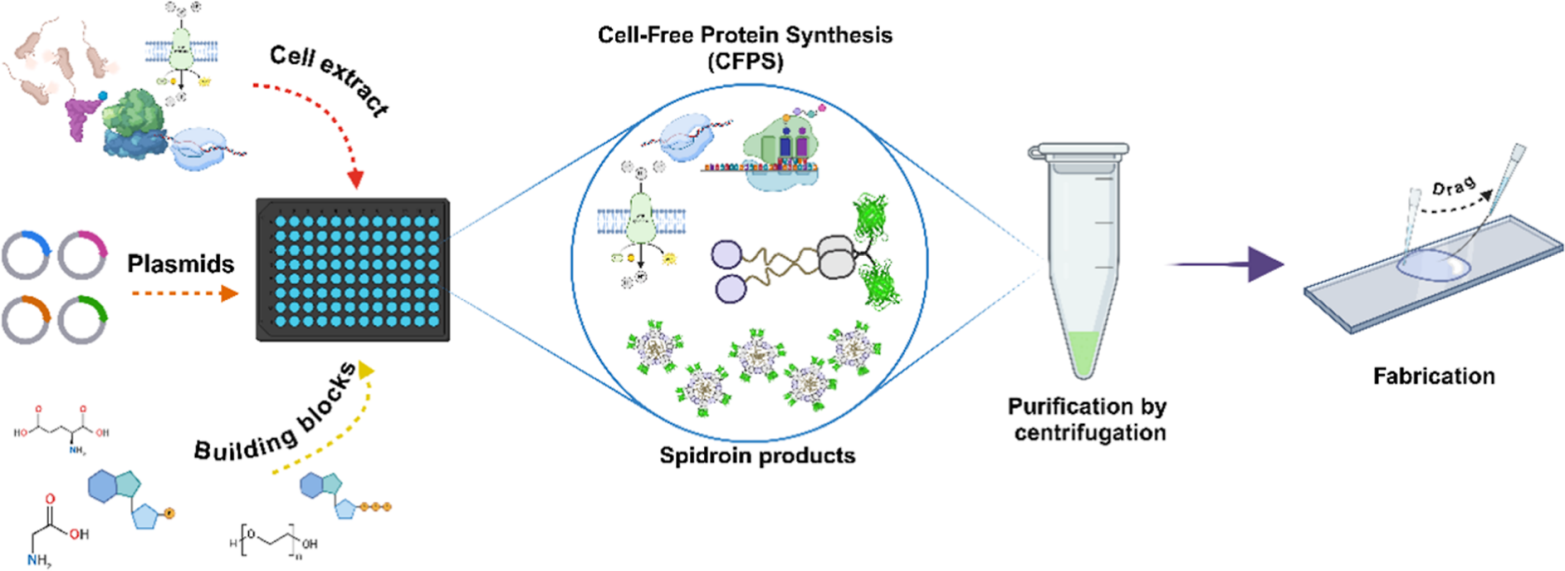

Spider silk spidroins, nature’s advanced polymers, have long hampered efficient *in vitro* production due to their considerable size, repetitive sequences, and aggregation-prone nature. This study harnesses the power of a cell-free protein synthesis (CFPS) system, presenting the first successful *in vitro* production and detailed characterization of recombinant spider silk major ampullate spidroins (MaSps) utilizing a reformulated and optimized*Escherichia coli* based CFPS system. Through systematic optimization, including cell strain engineering via knockout generation, energy sources, crowding agents, and amino acid supplementation, we effectively addressed the specific challenges associated with recombinant spidroin biosynthesis, resulting in high yields of 0.61 mg/mL for MaSp1 (69 kDa) and 0.52 mg/mL for MaSp2 (73 kDa). The synthesized spidroins self-assembled into micelles, facilitating efficient purification compared to *in vivo* methods, and were further processed into prototype silk fiber products. The functional characterization demonstrated that the purified spidroins maintain essential natural properties, such as phase separation and fiber formation triggered by pH and ions. This tailored CFPS platform also facilitates versatile cosynthesis and serves as an accessible platform for studying the supramolecular coassembly and dynamic interactions among spidroins. This CFPS platform offers a viable alternative to conventional *in vivo* methods, facilitating innovative approaches for silk protein engineering and biomaterial development in a high-throughput, efficient manner.

## 1 Introduction

The CFPS system is a versatile *in vitro* platform for biosynthesis and functional studies.^1^ Compared to *in vivo* production by hosts, CFPS provides a time- and labor-effective alternative for recombinant protein production.^2^ In addition, CFPS has been employed for a variety of applications such as high-throughput screening,^3−5^ transcription-translation system research,^6,7^ and the production of complex proteins that are traditionally challenging to synthesize *in vivo*.^1,8^ CFPS is particularly advantageous for expressing antimicrobial peptides,^9,10^ membrane proteins,^11,12^ and toxic proteins.^13,14^

Although CFPS provides substantial benefits, achieving high yields often requires optimization, particularly for challenging proteins such as those with high molecular weight (MW),^15^ disulfide bonds,^2,16,17^ or structurally associated membrane domains.^1,12,18^ In particular, many strategies, such as the genetic modification of host cells to enhance the biosynthesis activity of cellular extracts,^15,19^ the supplementation of energy sources,^20^ the use of crowding agents,^20^ or the use of mild surfactants,^21^ are all viable options for enhanced CFPS productivity. Numerous reports detail the large-scale production of proteins by CFPS methods, overcoming challenges associated with *in vivo* expression.^18,19,22,23^

The adaptability and tunability of CFPS present considerable opportunities for investigating the expression domains of novel and intricate biomacromolecules.^24^ This is particularly relevant for the synthesis of spider silk biomaterials, which are notoriously difficult to produce by using traditional methods. Spider silk materials are composed of spidroins characterized by their lengthy, repetitive, glycine-rich, and alanine-rich hydrophobic core sequences, flanked by hydrophilic N- and C-terminal domains (NTD and CTD), along with a tendency to form micelles.^25^ At present, the primary production approach via *in vivo* biosynthesis of recombinant spidroins faces several challenges, such as toxicity and metabolic burdens, constraints on product sizes, discrepancies in codon usage, randomly coiled hydrophobic cores, and development of inclusion bodies.^26−28^ This subsequently results in a typically poor yield and quality of recombinant spidroins during scaled production. Therefore, CFPS may provide an excellent alternative, bypassing the *in vivo* cellular obstacles and offering an innovative biosynthesis platform to replicate the intricate native processing of spidroins. This can be advantageous for creating an efficient system for the production of bioengineered spidroins.

However, there is currently limited research exploring the feasibility of producing silk or fibrous-associated proteins via CFPS, and few studies focus on relevant biomolecules with tandem repeats^29,30^ and codon-biased preferences.^31,32^ Therefore, it is worth further exploring CFPS in this research domain. In this pilot study, we seek to understand the following aspects: (1) the feasibility of spidroin production using CFPS, (2) the adaptability and tunability of CFPS in optimizing the yield and quality of the biosynthesized spidroin, and (3) the retention of functionality and structural integrity in the CFPS-produced spidroin. Upon this study, we aim to not only provide scientific insights into spidroin biosynthesis via CFPS but also offer technical advancements on cell-free synthetic biology and biomanufacturing of these future biomaterials or bioinspired polymers.

**Figure 1:**
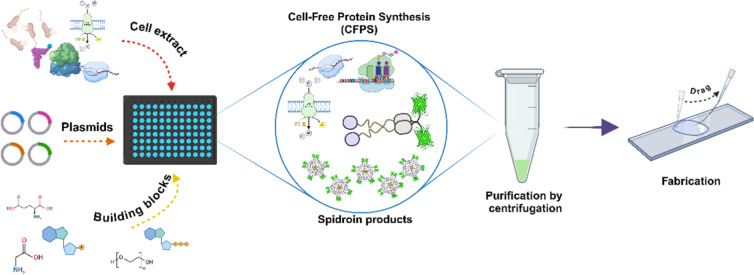
Spidroin production through the CFPS system with subsequent purification and characterization. Specifically, a mixture containing cell extract, plasmids, and other building blocks is added to a multiwell microplate for CFPS reaction. Synthesized spidroin products are purified and used for fabrication.

As depicted in Figure 1, this study presents the first successful *in vitro* synthesis and characterization of spidroins using CFPS. We have developed an*Escherichia coli* based CFPS system for the production of bioengineered major ampullate spidroins (MaSps), which are the main components of dragline spider silk. Our approach includes constructing a proprietary bacterial knockout library, serving as a tailored cell extract for CFPS formulation and efficiency tests. Through a systematic investigation of various factors, including the incorporation of crowding agents, the addition of energy sources, and strategic amino acid supplementation, we effectively improved the production of bioengineered spidroins. Subsequently, facile and time-effective purification of MaSp products is achievable through simple centrifugation-rinsing steps. Isolated MaSp products are characterized and spun into fibers, demonstrating the functionality of CFPS-synthesized spider silk materials. Furthermore, the CFPS system provides a versatile platform for probing multispidroin interactions and dynamics, as a feature that is difficult to achieve *in vivo*. This work highlights the potential for high-throughput applications in silk protein engineering and the development of novel biomaterials.

## 2 Results

### 2.1 Spidroin Production with Various Strain-Derived CFPS Cell Extracts

**Figure 2:**
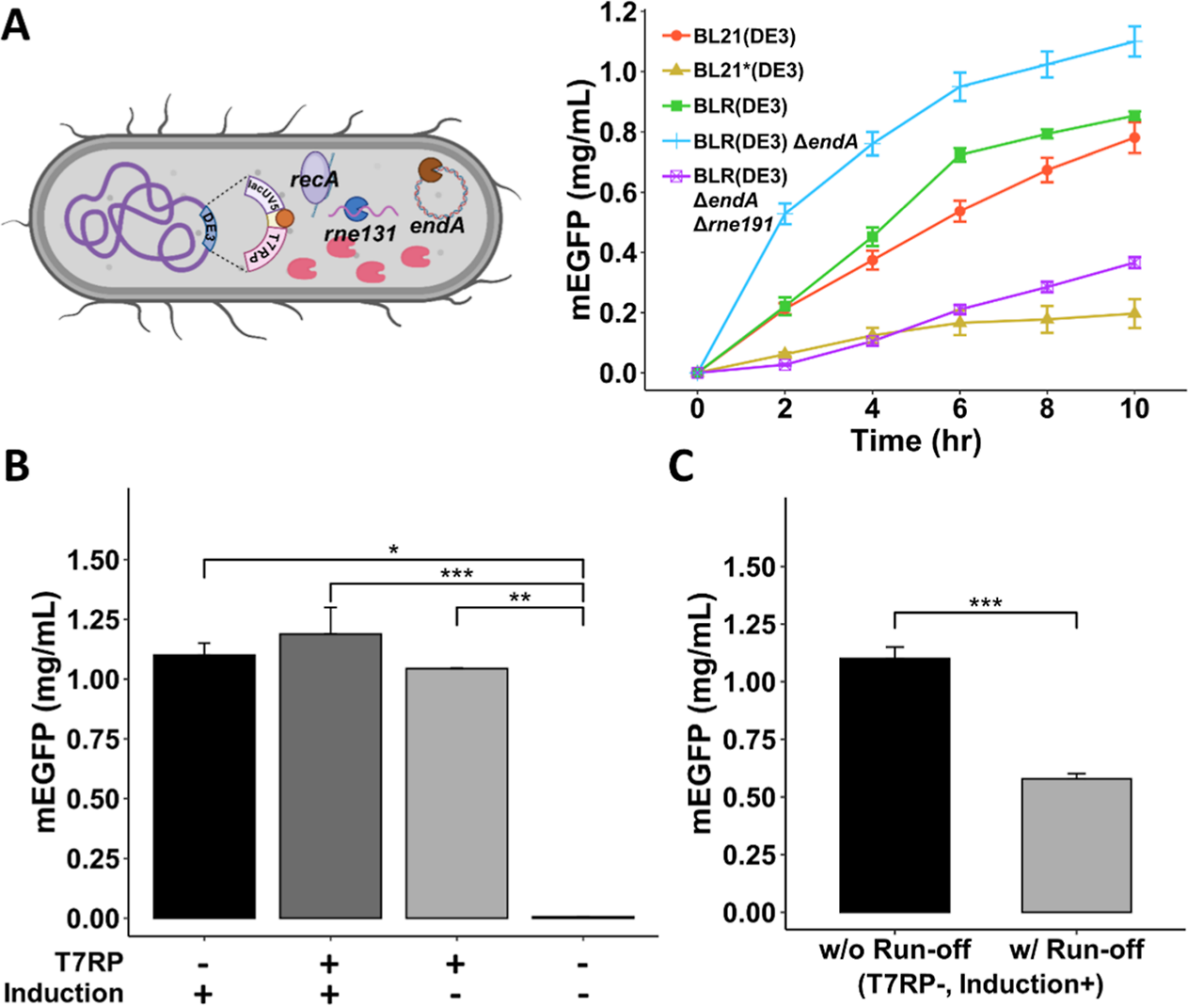
Tuning CFPS expression system by mEGFP synthesis. (A) A series of DE3-derived strains were constructed, including Δ*recA* (DNA recombination), Δ*endA* (endonuclease), and Δ*rne131* (RNA degradation) with the T7RP transcription system under an IPTG-inducible lacUV5 promoter. Five strains were evaluated for synthesis efficiency via mEGFP productivity (mg/mL): BL21(DE3), BL21*(DE3), BLR(DE3), BLR(DE3) Δ*endA*, and BLR(DE3) Δ*endA* Δ*rne131*. (B) Evaluation of exogenous and endogenous T7RP on CFPS production. T7RP + indicates supplementation of exogenous T7RP and induction + donates IPTG induction for endogenous T7RP synthesis. (C) Analysis of the cell extract runoff treatment (30 °C for 30 min) on mEGFP production by CFPS. All tests were conducted in triplicate; ns: nonsignificant, *p < 0.05, **p < 0.01, and ***p < 0.005.

To establish a cost-effective and productive CFPS platform, we have adopted a modified*E. coli*-based CFPS system (Cytomim).^17,33^ We first assessed the synthesis efficiency of the constructed CFPS system by producing the recombinant fluorescent protein mEGFP as a reference. Specifically, five*E. coli* DE3 strains, BL21(DE3), BL21*(DE3), BLR(DE3), BLR(DE3) Δ*endA*, and BLR(DE3) Δ*endA* Δ*rne131*, were utilized for generating the cell extract fraction for the CFPS system. Notably, the two knockout strains, BLR(DE3) Δ*endA* and BLR(DE3) Δ*endA* Δ*rne131*, were specifically created by our group using the λ-red recombination system^34^ and are not available elsewhere. All of the DE3 cells possess orthogonal transcriptional machinery through the use of T7 RNA polymerase (T7RP) following IPTG induction. As indicated in Figure 2A left, we hypothesized those knockouts are advantageous for enhanced productivity of CFPS, including Δ*rne131* for reducing mRNA degradation,^35^ Δ*recA* for reducing the chance of target DNA recombination,^36^ and Δ*endA* for enhanced plasmid integrity from endonuclease degradation.^37^ Specifically, for the cell extract-preparing procedure, all cell knockouts were grown at 37 °C shakers to an early log phase (OD_600_ ∼ 0.5), followed by 1 mM IPTG induction for inherent T7RP production until the OD_600_ reached 3.0. The cells were harvested and lysed to obtain the cell extract fractions for CFPS reactions subsequently.

The green fluorescence of mEGFP produced in the CFPS reactions using different cell extracts was quantified, functioning as a measure of the overall CFPS efficiency. In Figure 2A right, the plot illustrated the mEGFP productivity in various cell extracts, measured in relative fluorescence units (RFU) throughout the CFPS reaction. Overall, the CFPS with cell extract BLR(DE3) Δ*endA* yielded the highest mEGFP expression. The yield obtained was around 1.1 mg/mL at the 10 h reaction (as shown by the titration curve in Figure S2), which aligns with the results from other standard CFPS research studies.^17^ Additionally, BL21(DE3) and BLR(DE3) exhibited similar overall yields, while two Δ*rne131* strains (BL21*(DE3) and BLR(DE3) Δ*endA* Δ*rne131*) resulted in relatively low yields. Therefore, BLR(DE3) Δ*endA* performed the highest yield of mEGFP expression via the current CFPS reaction.

Subsequently, we sought to determine whether an additional supplementation of T7RP, as a transcriptional boost, to the cell extract can further favorably enhance the CFPS efficiency. T7RP was induced by IPTG in BLR(DE3) Δ*endA* harboring the vector pT7RP, followed by His-tag purification using a Ni-NTA column (Method S1). Figure 2B depicts the external supplementation of 50 μg/mL T7RP (referred to as T7RP+) with the endogenous production of T7RP by IPTG induction (denoted as Induction+). The incorporation of T7RP (T7RP+; Induction+) did not generate a significantly higher output compared to the IPTG induction alone configuration (T7RP–; Induction+), indicating that the endogenous T7RP present in the cell extract was enough for the CFPS response. The omission of both exogenous T7RP supplementation and endogenous T7RP synthesis (T7RP–; Induction−) led to the lack of mEGFP production. However, the introduction of exogenous T7RP (T7RP+; Induction−) may restore CFPS production to compensate for the deficiency of endogenous T7RP.

Furthermore, we also evaluated the effect of the runoff process, a preincubation strategy to eliminate the remaining nonspecific mRNAs present in the cell extract, on CFPS productivity.^38^ A 30 min incubation of cell extract at 37 °C was conducted initially, followed by the CFPS reaction using the treated cell extract.^19^ As the results demonstrated in Figure 2C, the mEGFP expression in the “w/Run-off” group showed a decrease in yield compared to the “w/o Run-off” group by ∼50% (***p < 0.005). Nevertheless, the runoff effect has been a topic of controversy, as it appeared to be unfavorable to the CFPS system we have developed and some other research.^39^ Considering overall convenience and expense, we excluded additional T7RP and runoff processes in the following spidroin production.

**Figure 3:**
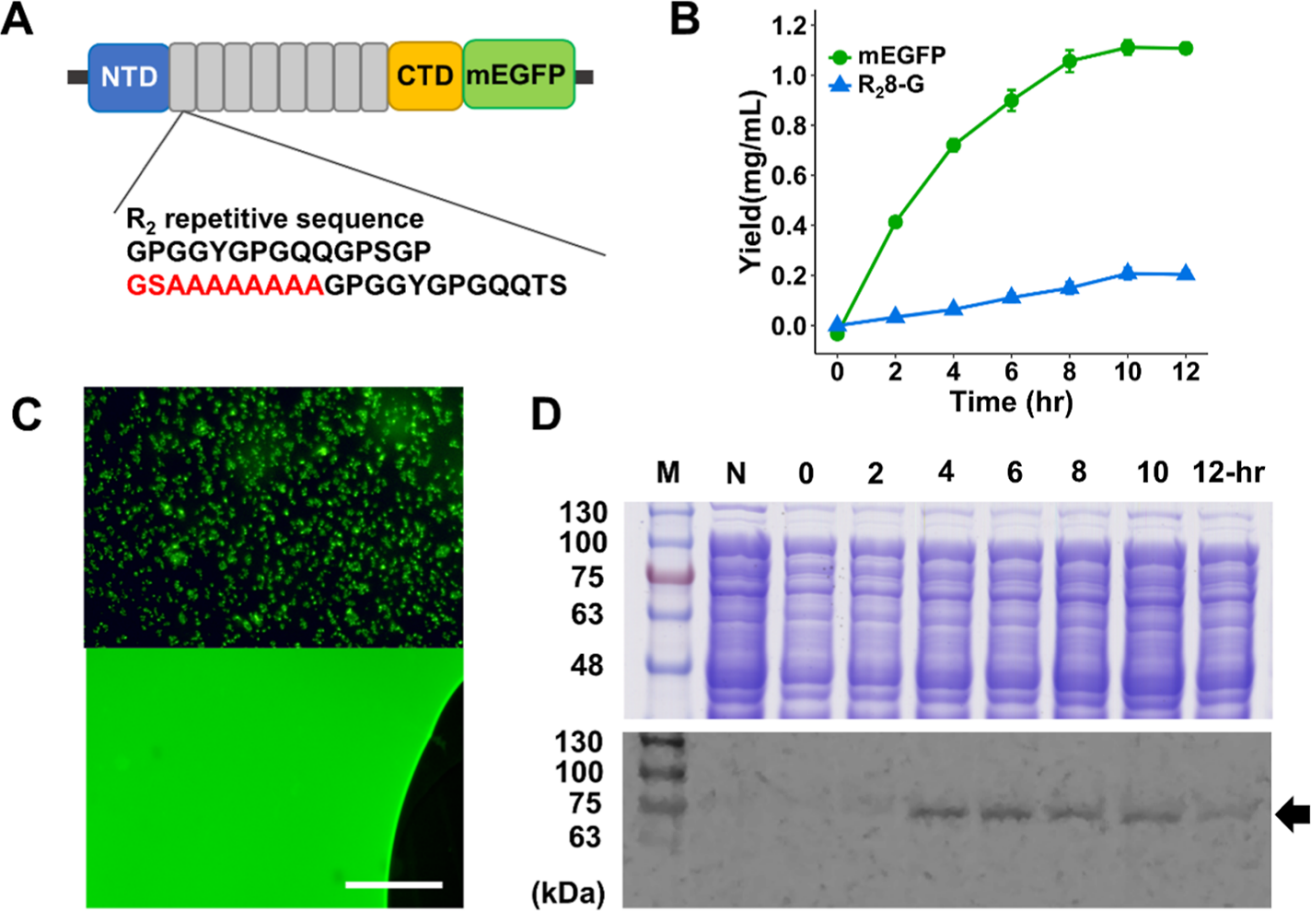
Production of spidroin R_2_8-G in the CFPS system. (A) Structure of the construct used for CFPS R_2_8-G production. R_2_ repetitive sequence was provided. β-Sheet forming amino acids are highlighted in red. (B) The temporal production yield of R_2_8-G and mEGFP via CFPS was determined. All tests were conducted in triplicate. (C) Microscopic analysis of CFPS-synthesized R_2_8-G and mEGFP. Upper image: R_2_8-G appeared in distinct particulate micelles in the CFPS mixture. Lower image: mEGFP in the CFPS mixture examined under the fluorescence microscope revealed a homogeneous green signal. Scale bar = 50 μm. (D) SDS-PAGE and Western blotting analyses of R_2_8-G in CFPS reaction. SDS-PAGE (upper plot) results for CFPS generation at varying synthesis durations; Western blotting (lower plot) for R_2_8-G detection via anti-EGFP (indicated by the arrow). M: protein marker. N: no-plasmid CFPS control group at *t* = 12 h.

Next, we sought to assess the potential of bioengineered spider silk synthesis within the CFPS platform. Briefly, we constructed a standard form of MaSp2 spidroin, which is derived from the dragline spider silk of *Nephila* spider.^31,40^ Illustrated in Figure 3A is the bioengineered spider silk construct containing 8 repeats of the core repetitive unit of MaSp2 (designated R_2_8), flanked by the NTD and CTD. Furthermore, mEGFP was integrated downstream of CTD, resulting in the final product NTD-R_2_8-CTD-mEGFP (R_2_8-G) for the following CFPS reaction (plasmid map in Figure S3).

Prior to proceeding with a comprehensive study of *in vitro* R_2_8-G production, we performed a prescreening of cell extracts from five DE3 strains (as in Figure 2) to confirm that our choice of strain was not biased by the mEGFP reporter. The R_2_8-G quantification was conducted using a titration curve (Figure S4) reported by Chen et al. (2023).^31^ As shown in Figure S5, BLR(DE3) Δ*endA* and BLR(DE3) displayed the top two expression yields of R_2_8-G, mirroring the trend observed with mEGFP expression (Figure 2). Specifically, the R_2_8-G yields were 0.21 mg/mL for BLR(DE3) Δ*endA* and 0.20 mg/mL for BLR(DE3). Interestingly, BLR(DE3) Δ*endA* displayed a slightly higher productivity of R_2_8-G than BLR(DE3) (though there was no statistical difference), with lower variability, indicating its robustness in CFPS expression. While Δ*endA* leads to endonuclease I depletion, theoretically enhancing plasmid integrity and reducing DNA fragmentation to favor full-length, functional mRNA, the fact that R_2_8-G productivity using BLR(DE3) Δ*endA* was not significantly higher than that using BLR(DE3) suggests that other CFPS factors may be limiting. Further investigation is warranted to fully explore the potential of BLR(DE3) Δ*endA*.

Next, in the time-course measurement as shown in Figure 3B, when using BLR(DE3) Δ*endA* as the extract source, the yield of R_2_8-G (blue curve) increased monotonically and reached a plateau signal after 8–10 h, resulting in a saturated yield of about 0.21 mg/mL. As a point of reference, the peak production of mEGFP, represented by the green curve, was 1.1 mg/mL. Subsequently, we examined the end products using a fluorescent microscope to validate the CFPS findings visually. Briefly, 2 μL of the CFPS reaction mixture producing R_2_8-G and mEGFP was pipetted onto a glass slide, covered with a coverslip, and then examined microscopically. As seen in Figure 3C, the fluorescence picture of the mEGFP group exhibited a uniform green color distribution throughout the slide; in contrast, R_2_8-G exhibited particulate formations with an approximate particle size of 700 nm, determined microscopically. R_2_8-G may form micelle-like particles owing to their amphiphilic characteristics,^25^ and these fluorescent particles were exclusively detectable after 4 h of CFPS reaction synthesis (Figure S6).

Additionally, molecular quantification of the R_2_8-G protein upon CFPS was also implemented by SDS-PAGE and Western blotting. The R_2_8-G signal was indiscernible in the SDS-PAGE analysis (upper plot) but became identifiable in the Western blot by anti-His staining (lower plot) after 4 h of CFPS reaction (Figures 3D and S7). The determined MW of R_2_8-G was around 75 kDa, referred to the corresponding protein marker, which aligns with the expected size of 73 kDa. The results also illustrate the viability of CFPS for the authentic production of spider silk protein.

To evaluate the performance of our CFPS system for R_2_8-G expression, we also included a commercial NEBExpress kit for comparison. As illustrated in Figure S8, our CFPS system rendered a similar overall expression level to the commercial kit at 10 h. While the commercial kit’s yield reached a plateau at 4 h, our system caught up at 8 h and reached a similar productivity level. The results demonstrated that our CFPS system, as well as the commercial kit, was capable of spidroin synthesis, and we aimed to enhance spidroin yield by further system modification for improved outcomes.^33^

### 2.2 Tuning the Yield of R_2_8-G Production via CFPS Reformulation

To enhance the yield of spidroins, we sought to re-evaluate the formulation of our Cytomim-based CFPS system. A series of additional reagents were incorporated to optimize the current CFPS system by adjusting the synthesis rate and yield: (1) crowding agents and detergents were utilized to minimize nonspecific interactions among spidroins and transcriptional/translational machinery, (2) redox reagents were employed to promote the rapid dimerization of spidroins through disulfide bond formation between different CTDs, and (3) energy sources and transcriptional/translational building blocks were incorporated to improve the synthesis machinery and efficiency. The production outcomes of the modified CFPS reactions are discussed in detail below.

#### 3.2.1 Triton X-100 Supplementation

Triton X-100 has been reported to improve the yield of antimicrobial peptides in the CFPS system^9^ and the initial reaction rate for GFP synthesis.^41^ For membrane proteins with large hydrophobic regions, the addition of detergents or lipids might be beneficial.^11^ Therefore, we supplemented four concentrations of Triton X-100 in the CFPS, including 0.5, 1.0, 10, and 50 CMC (critical micelle concentration, 1 CMC = 0.22 mM^42^).

Time-course production of R_2_8-G (RFU signal) in the CFPS indicated that Triton X-100 completely quenched the CFPS reaction (Figure S9A). Triton X-100 concentrations of 0.5 CMC and above yielded comparable results with no RFU signal detected relative to the blank. Given the probability that the detergent might jeopardize the inverted membrane vesicles essential for ATP synthesis via oxidative phosphorylation,^33^ we omitted Triton X-100 and other detergent assessments from our CFPS optimization.

#### 3.2.2 PEG8000 Addition

PEG8000 has been shown to enhance CFPS robustness at concentrations between 1.6% and 4% w/v.^43^ As a crowding agent, PEG8000 functions to improve the accessibility to macromolecules, as well as to simulate the environment of cytosol.^44,45^ In artificial spidroin production, we hypothesized that the addition of PEG8000 may increase the local concentration of R_2_8-G, therefore accelerating the rate of micelle formation.

**Figure 4:**
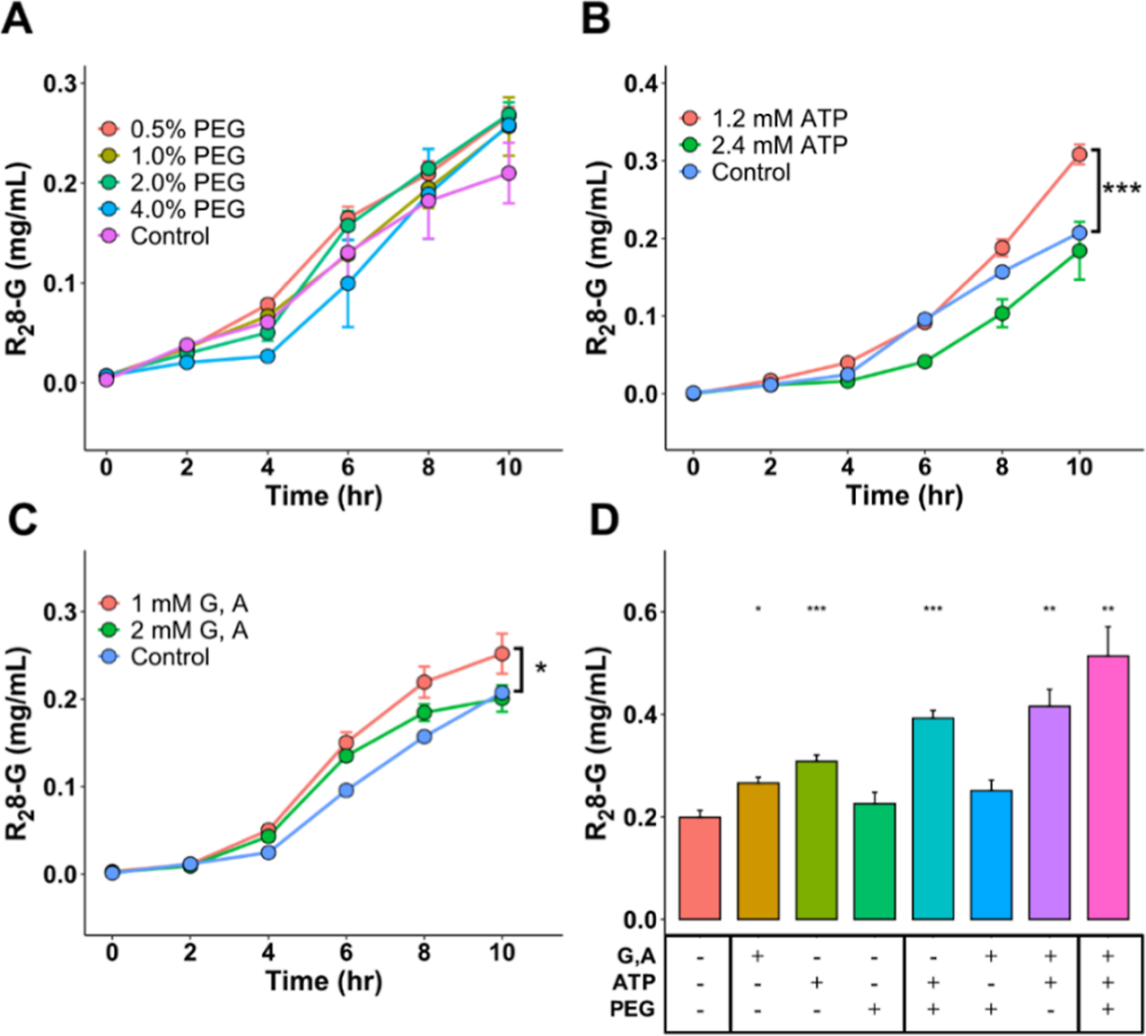
Optimization of spidroin production via CFPS reaction. (A–C) PEG8000, ATP, glycine, and alanine were added to improve the yield of R_2_8-G, with control as the group without any additive reagents. (D) A stepwise supplementation reaction was conducted by incorporating ATP, glycine/alanine, and PEG8000. The control group (G, A-, ATP-, and PEG-) served as the reference group. All tests were conducted in triplicate. *p < 0.05, **p < 0.01, and ***p < 0.005.

The addition of PEG8000 yielded minor increases in the yield of R_2_8-G (Figure 4A). Specifically, the supplementation of 0.5, 1, 2, and 4% PEG resulted in an enhancement of the final yield (10 h) at 10%, 5.8%, 10.7%, and 6.5%, respectively. Despite the absence of a substantial difference, we selected 2% PEG as the supplementary component in the CFPS optimization procedure.

#### 3.2.3 Glutathiol (GSSG) Supplementation

Glutathiol (GSSG), the oxidized variant of glutathione, was thought to enhance the yield when disulfide linkages were present in the produced protein.^19^ For instance, GSSG was included in the CFPS system for the production of antibodies and antibody fragments, resulting in enhanced yield.^17^ We anticipated that adding GSSG could affect disulfide bond formation in R_2_8-G, promoting spidroin production.

We included an additional 2 and 4 mM of GSSG at the initial stage of the CFPS reaction.^19^ While elevated GSSG levels may provide increased measurements at 2 h, the reaction was entirely inhibited in subsequent hours during the production of R_2_8-G (Figure S9B). Consequently, the supplementation of GSSG was omitted from CFPS optimization.

#### 3.2.4 ATP Supplementation

ATP functions as the primary energy source in the CFPS system, and ATP supplementation may enhance reaction rates and yields in some studies.^41^ Excessive ATP, however, can be detrimental, potentially through the overproduction of phosphate ions which may inhibit the CFPS reaction.^43^ To determine the optimal ATP concentration for our system, we tested two concentrations: 1.2 and 2.4 mM, in comparison to a control with no added ATP.^41^

The addition of 1.2 mM ATP resulted in a statistically significant (***p < 0.001) 46.8% ± 6.0% increase in the final yield at 10 h, compared to the no-ATP control. Conversely, 2.4 mM ATP showed only a minor, nonsignificant increase, suggesting that excessive ATP might inhibit the reaction (Figure 4B). Consequently, 1.2 mM ATP was selected for the optimized CFPS protocol.

#### 3.2.5 Inoculation with Premade Spidroin Micelles

Spidroin’s self-assembly into micelles is a complex process influenced by factors such as concentration and salt levels.^30^ At concentrations below its CMC, spidroin exists as soluble dimers.^25^ However, the exposed hydrophobic regions in these dimers can potentially interact with ribosomes during the CFPS reaction, potentially hindering translation and delaying micelle formation.^46^ We suspected that supplementing preformed spidroin micelles to the CFPS reaction might expedite micelle formation by reducing the time required for the process.

To test this hypothesis, we supplemented preisolated R_2_8-G spidroin micelles (at 0.05 and 0.10 mg/mL RFU) to our CFPS reactions. However, neither concentration led to a significant increase in the final yield at 10 h compared to the control group (Figure S9C). In fact, the higher dose (0.10 mg/mL) of spidroin micelle supplementation resulted in a lower yield than the control. This suggests that the addition of preformed spidroin micelles did not enhance the overall efficiency of the CFPS system under our conditions and was therefore excluded from the optimized protocol.

#### 3.2.6 Amino Acid Supplementation

Spider silk spidroins are rich in glycine and alanine residues, crucial for β-sheet formation.^31^ We hypothesized that supplementing the CFPS reaction with excess glycine and alanine might accelerate spidroin synthesis by ensuring a sufficient supply of these essential amino acids and potentially preventing depletion during the reaction. We tested the effects of providing 1 and 2 mM of each amino acid to the CFPS reaction.

The addition of 1 mM glycine and alanine increased the final yield by 20.0% ± 10.0% compared to the control (*p < 0.05). However, increasing the concentration to 2 mM resulted in a slight, nonsignificant decrease in CFPS yield (Figure 4C). Therefore, 1 mM supplementation of both glycine and alanine was incorporated into the optimized CFPS protocol.

#### 3.2.7 Stepwise Optimization on CFPS Reaction Conditions

Individual optimization tests showed that 2% PEG8000, 1.2 mM ATP, and 1 mM each of glycine and alanine are advantageous supplements to the CFPS process for spidroin production. We further addressed the enhancement by stepwise tests on each species (Figure 4D). In the individual supplementation tests, 1.2 mM ATP contributed the most significant role, while PEG8000 itself has a minor effect on the enhancement. Additionally, mixing ATP with glycine and alanine, or PEG8000, as supplements enhanced the yield 2.06- and 1.94-fold, respectively. Interestingly, the PEG8000 and glycine/alanine mixture did not significantly differ from the control group compared to the significant enhancement observed in the 1 mM glycine/alanine group.

Finally, these additives were integrated into a single reaction mixture and compared to the control without additives. In Figure 4D, a significant increase in R_2_8-G yield (∼2.5-fold, 0.52 mg/mL) was observed in the optimized group (ATP+, PEG+, G, and A+), validating the synergistic enhancement of spidroin production through this integrated optimization process (**p < 0.01).

### 2.3 Synthesis of R_1_8-R Spidroin Using the Optimized CFPS Condition

**Figure 5:**
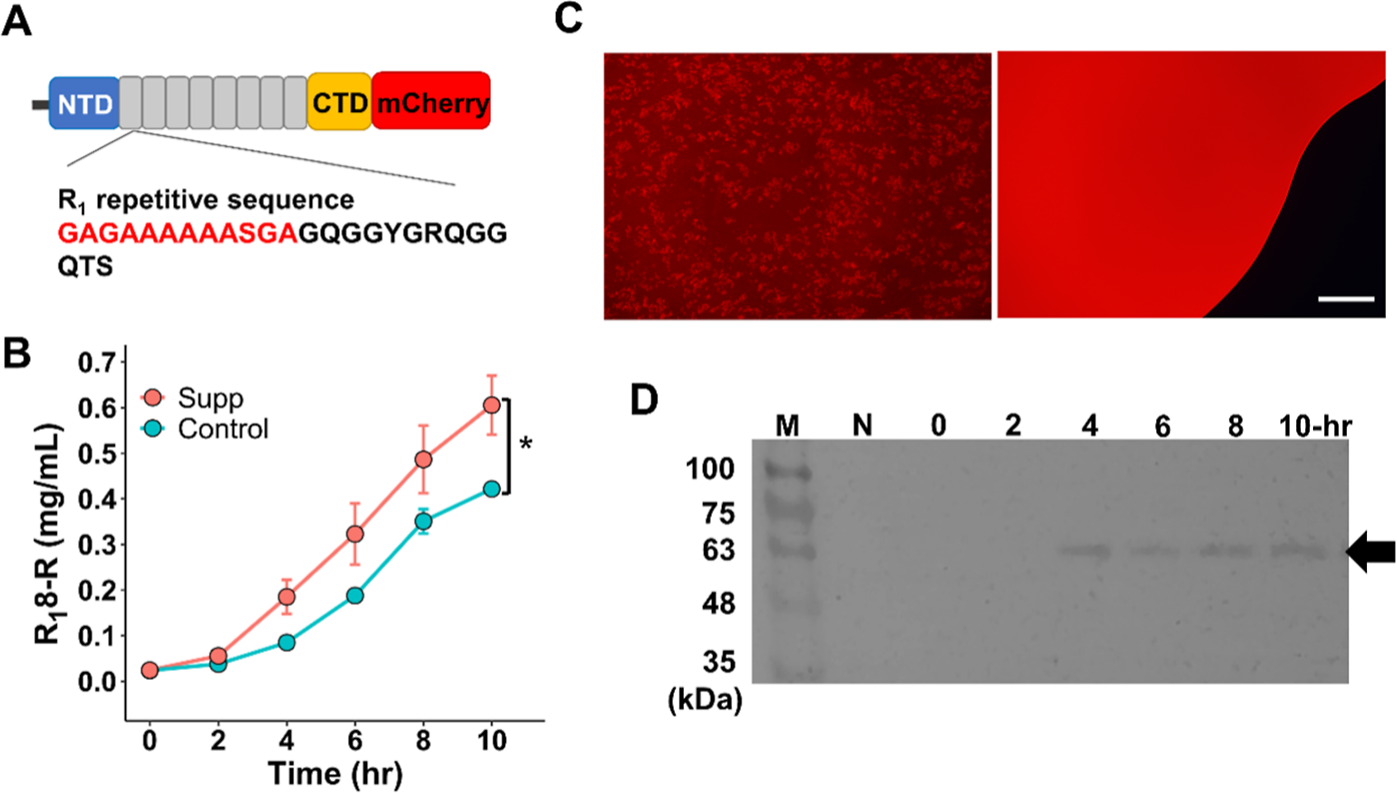
Production of spidroin R_1_8-R. (A) Structure of the construct used for CFPS R_1_8-R production. R_1_ repetitive sequence was provided. β-Sheet forming amino acids are highlighted in red. (B) The time-course production yield of R_1_8-R by the optimized CFPS setup (Supp) compared to the original CFPS setup (Control). CFPS experiments were conducted in triplicate. (*p < 0.05). (C) Left figure: R_1_8-R synthesized in the CFPS system displayed as distinct particles. Right figure: mCherry produced in the CFPS system was uniformly distributed. Scale bar = 50 μm. (D) Western blotting of R_1_8-R (indicated by the arrow) during the CFPS reaction. M: protein marker. N: no-plasmid control in the CFPS reaction.

To assess the versatility of our optimized CFPS system, we produced another spidroin variant, NTD-R_1_8-CTD-mCherry (R_1_8-R), utilizing a construct that includes the repetitive region R1 from *Nephila pilipes* MaSp1,^31,40^ with mCherry fluorescent protein substituted for mEGFP (Table S1). The quantification was similar to that of R_2_8-G, described in detail in Figure S10. As the CFPS reaction demonstrated in Figure 5A, the red fluorescence signal was utilized to represent the R_1_8-R expression levels. The yield enhancement for R_1_8-R with the optimized addition “Supp” was about 1.4-fold (0.61 mg/mL) than that of the “Control” group without additives (*p < 0.05). This also indicates the optimized CFPS system is suitable for producing a variety of spidroin proteins. After CFPS, the R_1_8-R products were examined under fluorescence microscopy (Figure 5B). R_1_8-R, similar to R_2_8-G, formed insoluble micelle-like particles (with a particle size of 500 nm), while the control mCherry protein displayed a homogeneous distribution of red signals. Furthermore, Western blotting confirmed the presence of R_1_8-R (hypothetical MW at 69 kDa) at the *M*_W_ ∼ 63–75 kDa after 4 h CFPS reaction (Figure 5C). Overall, the CFPS system shows great potential for the expression of spidroin proteins.

### 2.4 Spidroin Purification from CFPS Systems

**Figure 6:**
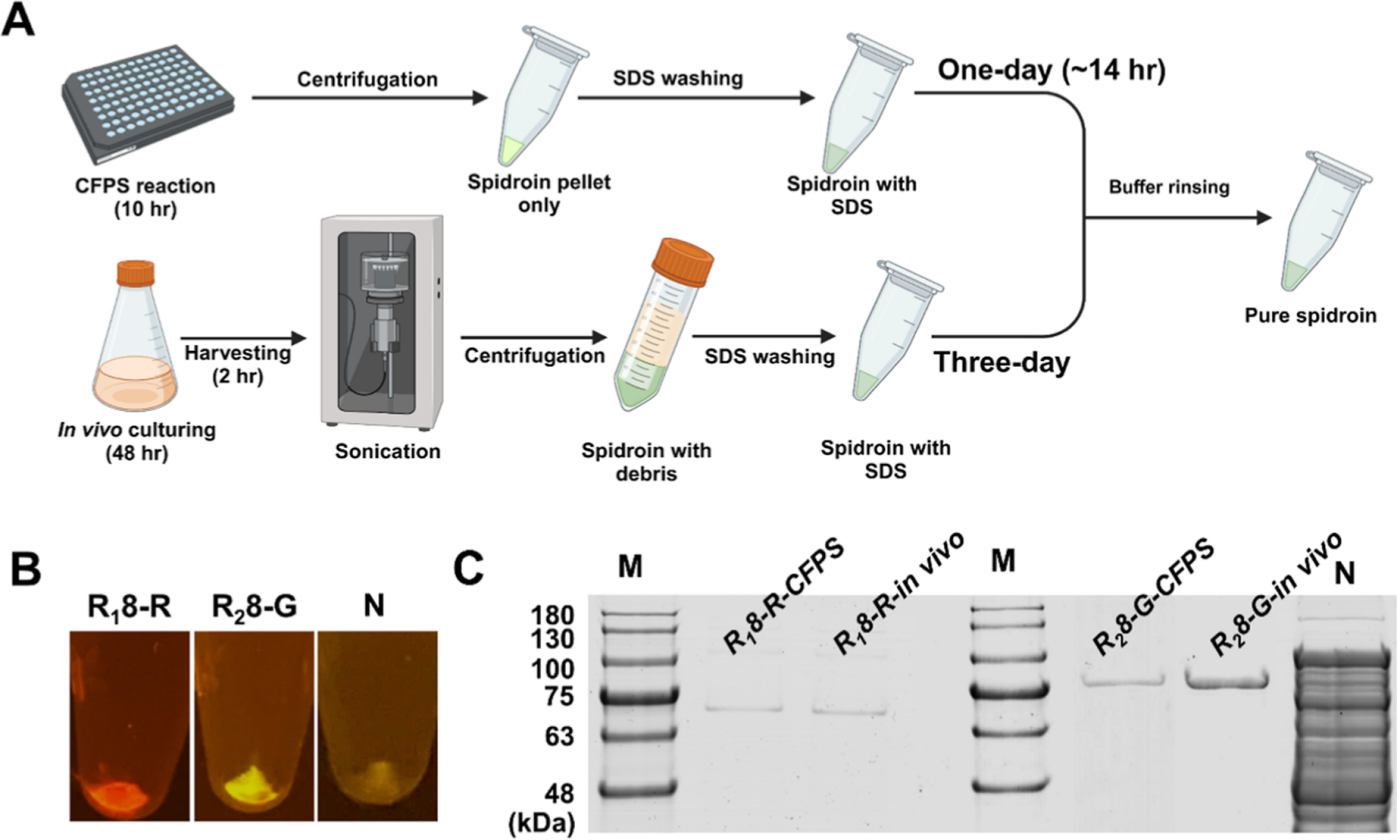
Purification of spidroin. (A) Workflow of spidroin production from CFPS reaction (upper) as a comparison to the traditional *in vivo* production (lower). The time duration for *in vivo* production and purification required 3 days. In contrast, the purification time for CFPS required 1 day. (B) Purified spidroins at 10 h. Spidroins existed as insoluble fluorescent pellets, while N (no-plasmid CFPS reaction) yielded no pellet. (C) SDS-PAGE analysis of purified spidroin R_1_8-R and R_2_8-G from both in vivo and CFPS reaction. M: protein marker. Negative control (N): the absence of spidroin plasmid in the CFPS and the lack of purification.

Given the adequate synthesis of spidroins in the CFPS setup, we subsequently sought to establish the purification processes enabling facile spidroin micelle isolation and characterization. As observed in Figures 3 and 5, spidroins produced through the CFPS system manifested as micellular structures, we refined the purification protocol outlined by Chen et al.(2023)^31^ Particularly described in Figure 6A, micellular spidroins products obtained after CFPS (6–10 h) were subjected to a simple centrifugation step to separate the crude spidroin pellet, thereafter undergoing washes with 0.5% SDS and rinses of ammonium phosphate (NH_4_Pi) buffer (0.5 M; pH 8.0) for 2–3 h. Such rapid purification of CFPS has the potential to eliminate the extended cell culturing and lysis steps associated with conventional *in vivo* production techniques (up to 3 days). Consequently, CFPS presents a promising strategy for spidroin purification that demonstrates a significant cost-effectiveness.

As depicted in Figure 6B, CFPS-purified R_1_8-R and R_2_8-G existed as insoluble pellets with their corresponding fluorescence, while the no-plasmid negative control (N) exhibited a negligible pellet after the same purification process. The outcome demonstrated the viability of isolating spidroins in CFPS using a simple purification method. The purified R_1_8-R and R_2_8-G were subsequently analyzed by using SDS-PAGE (Figure 6C). Both the CFPS and *in vivo* spidroins exhibited clear and singular products postpurification, displaying the required sizes of 69 kDa and 73 kDa for R_1_8-R and R_2_8-G, respectively. The control land (N) denotes the blank CFPS response devoid of plasmids in the CFPS reaction. The SDS-PAGE analysis demonstrates that the high purity of spidroin products strongly supports the notion that CFPS facilitates the synthesis of purifiable and quantifiable spidroins, which is advantageous for spider silk material research.

To further characterize the size distribution of R_2_8-G micellular particles and their response to different solvent conditions, we subjected purified R_2_8-G to a dynamic light scattering (DLS) analysis. Prior to the analysis, we resuspended the pellet in Milli-Q water and NH_4_Pi (0.5 M, pH 8.0). Fluorescence microscopy revealed that R_2_8-G particles within Milli-Q were smaller and more soluble (based on the background fluorescence) compared to NH_4_Pi (Figure S11A). DLS analysis of R_2_8-G in Milli-Q water showed micelle formation at ∼335 nm, while in NH_4_Pi, the predominant size was ∼755 nm. The respective polydispersity index (PDI; *D̵*) was 0.48 and 0.50, indicating a relatively broad and heterogeneous size distribution, typical of self-assembled protein structures (Figure S11B,C). This increase in particle size in NH_4_Pi is consistent with our microscopic observations of enhanced aggregation and liquid–liquid phase separation (LLPS)^47^ under these conditions. Similarly, natural spidroin micelles also display a spectrum of particle sizes from 200 to 800 nm, indicating multilayered micellar structures.

### 2.5 Characterization of CFPS-Produced Spidroin

To evaluate the responsiveness of CFPS-generated spidroins for material applications, we examined their self-assembly behavior under diverse circumstances, focusing on the LLPS aspects and fiber production.^31,46^ Mimicking the natural process of spider silk production, spidroins have been seen to create condensed droplets (as a manifestation of LLPS) in an ionic solution rich in phosphates.^47^ Furthermore, in acidic circumstances, the NTDs of spidroins undergo dimerization, prompting β-sheet formation within the repeated units and eventually resulting in fibrilization of spidroin and fiber formation with shear force.^31,46−49^ Consequently, we introduced the spidroin variants (R_1_8-R and R_2_8-G) purified from CFPS to various pH and ionic settings to assess their self-assembly potential and fiber formation capability.

**Figure 7:**
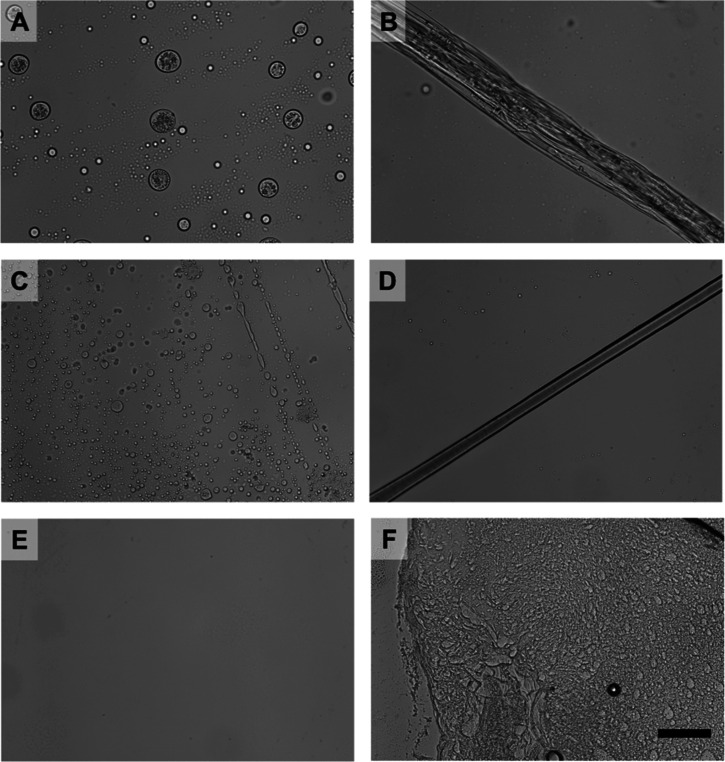
Characterization of LLPS and fiber formation for CFPS-produced spidroins. (A,B) LLPS effect and fiber formation of R_2_8-G at pH 8 and pH 4.3. (C,D) LLPS effect and fiber of R_1_8-R at pH 8 and pH 4.3. (E) R_2_8-G under the Milli-Q water. (F) HFIP-dissolved bovine serum albumin (BSA) in pH 4.3. Scale bar = 50 μm.

First, the molecular behaviors and fabrication capability of purified CFPS-produced R_2_8-G were evaluated. Briefly, the micellar R_2_8-G was preconditioned in hexafluoro-isopropanol (HFIP), forming a homogeneous spidroin solution. Subsequently, upon injecting into the NH_4_Pi bath at pH 8, the LLPS effect of R_2_8-G was observed (Figure 7A), characterized by condensed droplets of varying sizes that gradually fused. Upon transitioning to the NH_4_Pi bath at pH 4.3, a clear single fiber was noted along the microtip, being dragged from the buffer (Figures 7B and S13A). The resulting ribbon-like fiber displayed nonuniform morphology and size variation, ranging from 40 to 50 μm.

Furthermore, we aimed to investigate if CFPS-synthesized R_1_8-R, distinct from R_2_8-G in its repetitive unit, is capable of forming fibrils in reaction with pH gradients and varying ionic conditions. As shown in Figure 7C,D, R_1_8-R exhibited a reaction similar to that of R_2_8-G, both of which experienced LLPS at pH 8.0 (Figure 7C) and fibrilization at pH 4.3 (Figure 7D). In contrast to R_2_8-G, the single fiber produced by R_1_8-R exhibited a more uniform appearance under microscopic observation, with a diameter of around 30 μm.

Additionally, we developed an NTD-derived variant of R_2_8-G, designated NDCR-R_2_8-G, which has two amino acid changes (D36K and K61D) in the NTD.^50−52^ The CFPS-synthesized NDCR-R_2_8-G exhibited the ability for LLPS in NH_4_Pi buffer (pH 8.0), while it displayed a less cohesive and more dispersed fibril formation pattern relative to R_1_8-R and R_2_8-G (Figure S13B,C) in an acidic condition (pH 4.3) with shear stress applied. The mutations of amino acids in the NTD are significantly linked to disruptions in dimerization and fiber assembly.^50^

These behaviors are critically dependent on specific ionic conditions; injecting spidroins R_1_8-R and R_2_8-G in Milli-Q water alone did not induce LLPS or fiber formation (Figures 7E and S14). In comparison, under a higher acidic ion bath (0.5 M NH_4_Pi pH 2.8), R_1_8-R and R_2_8-G can also form fibers, although the texture showed some differences, exhibiting a less aligned and compact morphology, compared to fibers at the bath of pH 4.3 (Figure S14). The unique self-assembly properties of spidroins are distinct from those of other globular proteins such as BSA. Introducing HFIP-dissolved BSA (as the control material) to an acidic buffer (pH 4.3) only formed aggregates, without any evidence of LLPS or fiber formation (Figure 7F). This confirms that CFPS-produced spidroins recapitulate the characteristic self-assembly of natural spider silk proteins, demonstrating the potential for tailoring their structures and properties through the controlled manipulation of the external environments.

### 2.6 Cosynthesis of Spidroins via CFPS

Additionally, we sought to evaluate the feasibility of cosynthesizing various spidroin variants to create complex spider silk analogues that replicate the natural composite structure of spider silk^53^ through the simultaneous coexpression of R_2_8-G and R_1_8-R spidroins in the same CFPS reaction, while examining the intermolecular interactions and structural hierarchy. We specifically coexpressed R_2_8-G and R_1_8-R in the same CFPS reaction (CoR_1_-R_2_) by combining two plasmids in a 1:1 molar ratio, maintaining the overall plasmid quantity at 1 μg per 100 μL.

**Figure 8:**
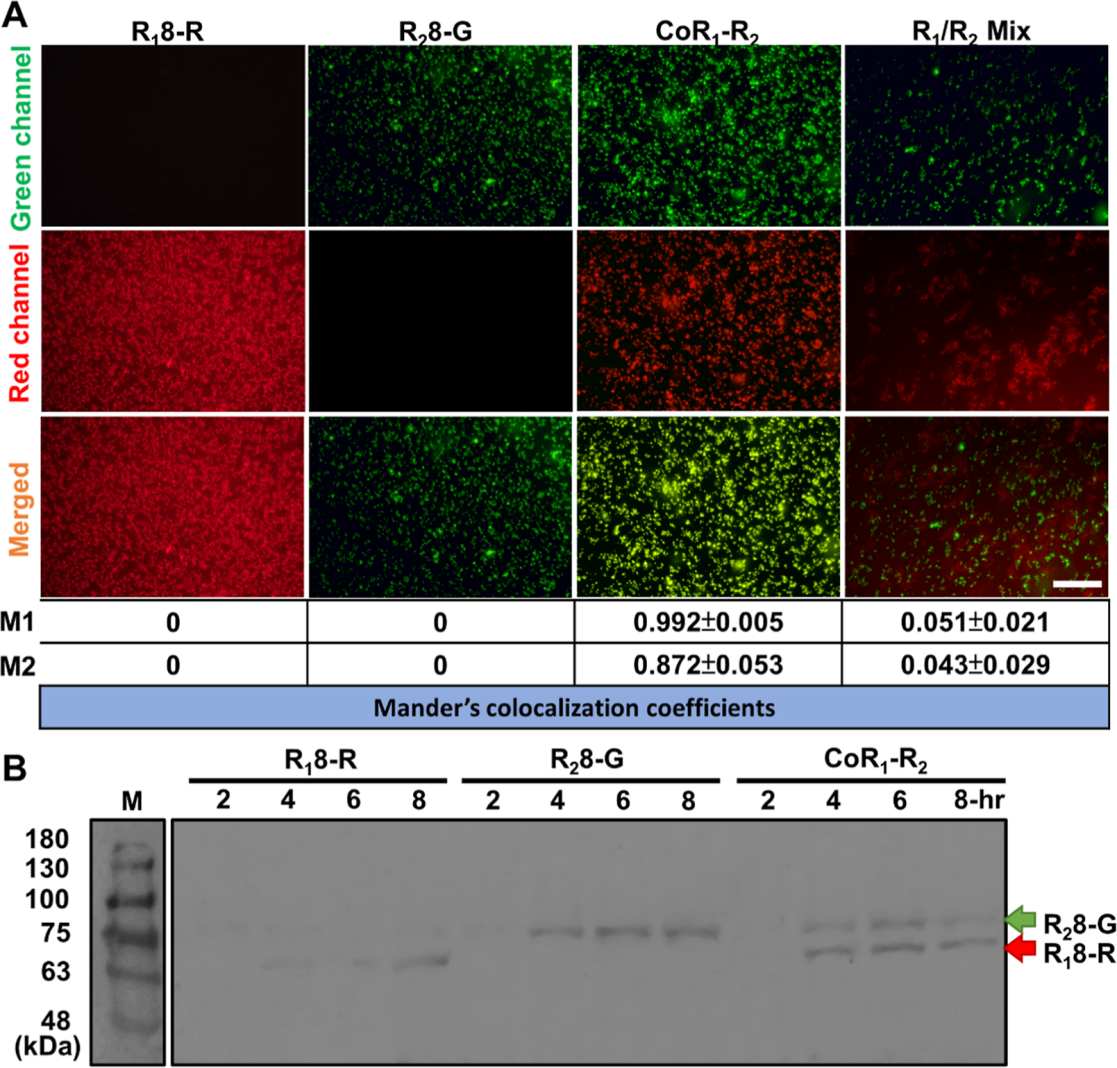
Coexpression of R_1_8-R and R_2_8-G. (A) Examination of synthesized spidroin under fluorescence microscopy. In the CoR_1_-R_2_ group, both R_1_8-R and R_2_8-G plasmids were concurrently integrated into the same CFPS reaction for R_1_-to-R_2_ cosynthesis, showing robust colocalization of green and red signals. For the postpurification mixture (R_1_/R_2_ mix) group, R_1_ and R_2_ were first synthesized independently and then mixed together. The resulting fluorescence exhibited noncolocalization, with R_1_8-R and R_2_8-G displaying only red and green signals, respectively. Mander’s coefficients: M1 represents the red-to-green signal, whereas M2 denotes the green-to-red signal. Scale bar = 50 μm. (B) Western blotting results of R_1_8-R (red arrow), R_2_8-G (green arrow), and CoR_1_-R_2_ in a time series CFPS reaction. M: protein marker.

Upon coexpression in Figure 8A, the sample CoR_1_-R_2_ generated hybrid micelles in the CFPS reaction, as seen by the amalgamation of the red and green fluorescence signals, yielding strong yellowish signals when merged. Conversely, simply combining presynthesized R_1_8-R and R_2_8-G resulted in distinct micelles with no colocalization of red and green signals (without no apparent yellow signals when merged). In the two distinct control groups, R_1_8-R and R_2_8-G each exhibited sole red and green signals, respectively. Microscopic analysis of the CoR_1_-R_2_ group revealed a high degree of colocalization, with Mander’s coefficients of 0.992 (M1; red-to-green signal) and 0.872 (M2; green-to-red signal), while the *R*_1_/*R*_2_ mix group displayed minimal colocalization (M1 = 0.051 and M2 = 0.043). These results demonstrate that coexpression facilitates the formation of mixed micelles, whereas simply mixing the two spidroins leads to separated distribution. In contrast to *in vivo* systems, where purification steps can disrupt the morphology of different spidroins, CFPS allows for the observation of R_1_8-R and R_2_8-G interactions in real time, providing more accurate outcomes for probing the multispidroin interactions.

Furthermore, RFU measurement validated the productivity of R_1_8-R and R_2_8-G (Figure S15). Additionally, Western blotting revealed the presence of two different bands, corresponding to R_1_8-R (red arrow) and R_2_8-G (green arrow) at 73 and 69 kDa, respectively, in the CoR_1_-R_2_ group (Figure 8B), while only a single band product was identified in each control group. This also provides further evidence confirming the viability of multispidroin production in CFPS as an effective strategy. This can offer an efficient prototyping framework for the future fabrication of adjustable and customizable spider silk biomaterials.

## 3 Discussion

In this study, we have successfully developed and reformulated an *E. coli*-based CFPS system for the *in vitro* production of bioengineered spider silk spidroins. Our key findings include the achievement of high spidroin yields, the demonstration of functional self-assembly, and the development of a versatile platform for cosynthesis and interaction studies. Through a systematic investigation and optimization of various parameters, including cell extract selection and engineering, additive supplementation (PEG, ATP, glycine, and alanine), and reaction conditions, we significantly boosted the yield of spidroin synthesis. The optimized CFPS system produced both R_2_8-G and R_1_8-R functional spidroins, enabling material fabrication into prototype fiber products. This research provides not only scientific insights but also technical advancements in biomaterial applications.

The Cytomim system serves as the foundation of our CFPS approach, providing a cost-efficient and scalable manufacturing method.^33^ Despite the modified CFPS procedures that include extra components such as costly ATP sources, they remain more economical compared to alternative CFPS platforms that solely rely on expensive energy systems such as creatine phosphate and phosphoenolpyruvate.^54^ Furthermore, our modified CFPS procedures and each supplement could synergistically enhance spidroin synthesis. However, we acknowledge that further research is needed to fully elucidate the underlying mechanisms of this synergistic effect and to optimize the system for even higher yields.

Additionally, our reformulated CFPS system demonstrated highly competitive yields of 0.52 g/L for R_2_8-G and 0.61 g/L for R_1_8-R, outperforming the yields of the commercial reaction kit (Figure S9) and many other batch CFPS systems, particularly for difficult-to-express and tandem proteins.^15,34,55^ Typically, spidroin production employs *in vivo* techniques (using flasks and bioreactors), resulting in yields of 0.0025–0.663 g/L (batch) or 0.015–3.6 g/L (fed-batch), although these processes are time-consuming (up to 40 h) as seen in Table 1. Our CFPS method attains yields that are typically equivalent while drastically reducing synthesis time to 10 h, resulting in an improved production rate (up to 0.061 g L^–1^ hr^–1^). The enhanced production rate and streamlined workflow, together with its elevated productivity (Table 1), clearly indicate that CFPS represents a viable method for spider silk production.

**Table 1:** Summary of Spidroin Production and Yield Comparison

Production mode	Spidroin	Size (kDa)	Time (hr)	Production yield (g L^–1^)	Production rate (g L^–1^ hr^–1^)	ref
Batch	MaSp1	10–28		0.0025–0.015		(56)
	A1S8_20_	57.64		0.01		(57)
	MaSp1s	47.2		0.4		(46)
	ADF3	56.2		0.256		(58)
	MaSp1	65–163		0.663		(59)
Fed-batch	96-mer	284.9	24	0.5	0.020	(27)
	CRGD-15mer	48		0.015		(60)
	MaSp2	120	36.7	0.65	0.017	(61)
	192-mer	556	24	2	0.08	(62)
	MaSp2	202	40	3.6	0.09	(63)
CFPS	R_1_8-R	69	10	0.61	0.061	This study
	R_2_8-G	73	10	0.52	0.052	This study

To further demonstrate the versatility of our CFPS system and expand its capacity for producing high-molecular-weight spidroins, we attempted to synthesize larger spidroin species, NDCR-R_1_32-CTD-mEGFP (117 kDa) and NDCR-R_1_128-CTD-mEGFP (314 kDa), containing 32 and 128 repeats of R_1_, respectively. Preliminary experiments demonstrated successful production, as evidenced by fluorescence readings and microscopic visualization (Figure S16). Fluorescence microscopy revealed the formation of micellular particles after 10 h of CFPS, while the overall production yield is to be further validated; the results demonstrate the potential of our CFPS system for synthesizing high-molecular-weight spidroins (up to 117 ∼ 314 kDa), opening new avenues for creating more complex and functional biomaterials.

Last but not least, CFPS presents a compelling opportunity to produce spider silk on a large scale, owing to its scalability.^19^ Advancements in synthetic biology and biotechnology could revolutionize the bioproduction landscape of CFPS in the future, potentially competing with the existing *in vivo* fermentation paradigm.^2^ Key factors to achieve scalable CFPS production include maintaining operation at larger reaction volumes, developing continuous-flow systems to sustain substrate chemostasis and remove inhibitory wastes, and automating the purification process.^38^ For example, a recent breakthrough of a continuous-exchange cell-free system (CFCE) signifies a transition from conventional batch processes of CFPS into a continuous production mode, facilitating prolonged biosynthesis.^64−66^

Moreover, we sought to broaden the application of our CFPS system in high-throughput screening of multispidroin interaction (Figure 8), enabling the design of novel hybrid materials that mimic native spider silk or other bioinspired composites requiring complex coassembly, a feature that is difficult to achieve using *in vivo* systems. Ultimately, we envision that our innovative efforts will enhance research in the design and scalable manufacture of novel biomaterials, leading to tangible advancements in the field.

## 4 Materials and Methods

### 4.1 Plasmid Construction

Plasmid pCFPS was constructed from the vectors, pUC57 and pET28a.^31^ Briefly, the T7 promoter, ribosome binding site, open reading frame (with 6xHis), and T7 terminator sequences were amplified from pET28a, and the *lacO* sequence was mutated via PCR. For constructing pCFPS-mEGFP and pCFPS-mCherry, the mEGFP sequence and mCherry sequence were PCR-amplified, flanking with *Nde*I and *Hin*dIII cloning sites at the ends, followed by further insertion to pCFPS using *Nde*I and *Hin*dIII double digestion and T4 ligation processes.

Artificial spidroin R_1_ and R_2_ sequences were extracted from MaSp1 and MaSp2 of *Nephila* spiders.^31,40^*E. coli*-codon optimized R_1_ and R_2_ sequences and the replication of the repeats were obtained as previously described.^31^ The full sequence of R_1_ and R_2_ was described in Table S1. NTD-R_1_8-CTD and NTD-R_2_8-CTD were inserted into the cutting site *Nde*I and *Hin*dIII on plasmid pCFPS. mCherry and mEGFP were inserted downstream of the CTD fragment. For constructing pCFPS-NDCR-R_2_8-G, two mutations on NTD (D36K, K61D) were introduced by site-directed mutagenesis using PCR. Mutated charge-reversed NDCR was used to replace the original NTD sequence by double digestion (*Nde*I and *Nhe*I), followed by T4 ligation procedures.

Plasmid pT7RP originated from pQE80 with the kanamycin resistance gene replaced by the chloramphenicol resistance gene. *t7rp* gene was obtained from BLR(DE3) through PCR amplification with *Bam*HI- and *Sal*I-flanked primers and inserted into pQE80 through double digestion and T4 ligation.

### 4.2 Cell Extract Preparation

Cell extracts of *E. coli* BL21(DE3), BL21*(DE3), BLR(DE3), BLR(DE3) Δ*endA*, and BLR(DE3) Δ*endA* Δ*rne191* were prepared by the following procedure.^39^ Cells were first inoculated from frozen stocks to 5–10 mL of LB medium in the culture tubes and incubated at 37 °C, 250 rpm overnight. The next day, the overnight culture was reinoculated to different volumes (200 and 1000 mL) of 2× YPTG medium (5 g of NaCl, 16 g of tryptone, 10 g of yeast extract, 7 g of potassium phosphate monobasic, 3 g of potassium phosphate dibasic, and 18 g of d-glucose/1000 mL, pH 7.2) in the baffled flasks at the seeding density of OD_600_ ∼ 0.01. Cells were later incubated at 37 °C and 250 rpm until the OD_600_ reached 0.5, and 1 mM IPTG was added to induce the endogenous T7RP expression. Cells were harvested by centrifugation (8000 g, 15 min, 4 °C) once OD_600_ reached 3.0, followed by triple washes with S30 buffer (10 mM Tris-acetate, 14 mM magnesium acetate, 60 mM potassium acetate, and 2 mM dithiothreitol, pH 8.2). The washed cell pellet was weighed (wet cell weight, WCW), rapidly frozen in liquid nitrogen, and stored at −80 °C.

For the lysis process, 1 g of WCW of cells was resuspended in 1 mL of S30 buffer. The suspension was sonicated on ice (5.5 W, 30 pulses, 3s on/5s off, and 60% amplitude). The suspension was then centrifuged at 12,000 g for 10 min at 4 °C, and the supernatant fraction was collected. For the runoff reaction, the supernatant of the cell extract was incubated at 37 °C and 250 rpm for 30 min. Afterward, the cell extract samples were centrifuged again at 4 °C and 10,000*g* for 10 min. The supernatant was collected, immediately frozen in liquid nitrogen, and stored at −80 °C until needed.

### 4.3 CFPS Reaction

The standard CFPS reaction was performed under the following conditions: 30 μL of cell extract was mixed with 25 μL of CFPS master mix and 1 μg of the reaction plasmid (obtained using the ZymoPureII Plasmid MidiPrep Kit), and the final volume was adjusted to 100 μL by Milli-Q water, unless otherwise specified. The CFPS reaction mixture, adopted from Cai et al. (2015),^17^ was formulated as follows: 1.2 mM AMP, 0.86 mM GMP, CMP, UMP, 1 mM tyrosine, 2 mM other amino acids, 8 mM magnesium glutamate, 260 mM potassium glutamate, 4 mM potassium oxalate, 15 mM potassium phosphate at pH 7.0, 1.5 mM spermidine, and 2 mM GSSG. Supplemental T7RP (purification process described in Method S1) was introduced at a concentration of 50 μg/mL if required. For the tests of additives, the supplements were solubilized in Milli-Q water and were included in the CFPS reaction mixture. For the CFPS reaction using the commercial kit (NEBExpress, New England Biolabs), the reaction was conducted according to the supplier’s instructions.

For the CFPS reaction setup, 100 μL of the CFPS reaction mixture was pipetted into a black 96-well plate. Unused wells were filled with Milli-Q water to prevent evaporation. The plate was sealed with an AeraSeal film (Excel Scientific) and placed on an orbital shaker at 1000 rpm in a humidified chamber. The reaction temperature was maintained at 30 °C, and the reaction was monitored by the fluorescence measurement using a SpectraMax M2^e^ microplate reader (Molecular Devices) at bihourly basis. The excitation/emission wavelength for mEGFP was 485/525 nm, and the excitation/emission wavelength for mCherry was 587 nm/610 nm.

### 4.4 *In Vivo* Spidroin Production

Plasmids pCFPS-R_2_8-G and pCFPS-R_1_8-R were introduced individually into manufactured *E. coli* BLR(DE3) Δ*endA* competent cells using heat-shock transformation. Single colonies were picked and inoculated into 5 mL of LB medium with 100 μg/mL ampicillin in the culture tube. The culture was incubated at 37 °C and 250 rpm overnight (∼16–18 h). Overnight cultured cells were refreshed into fresh LB medium (200 mL in flask, 100 μg/mL ampicillin) at 1% reinoculation ratio (seeding density at OD_600_ ∼ 0.01) and cultured at 37 °C, 250 rpm. After the OD_600_ reading reached 0.5, 1 mM IPTG was included to induce the production of spidroin proteins, with the temperature shifted to 25 °C for 16–18 h.

### 4.5 Purification of Artificial Spidroin

For the purification of CFPS-synthesized spidroin products, the CFPS reaction mixture was gathered and centrifuged at 20,000*g* for 10 min. The pellet was rinsed with a 0.5% SDS solution in 50 mM Tris–HCl buffer (pH 8.0) to isolate spidroins from other precipitates. Washed spidroin was centrifuged at 20,000*g* for 10 min and further rinsed with 0.5 M NH_4_Pi buffer (pH 8.0) multiple times until no visible bubbles were observed after a brief vortex. The purified spidroin was kept at 4 °C for short-term storage or −80 °C for long-term preservation.

For the purification of *in vivo* produced spidroin, the cell pellet was gathered by centrifugation at 8000*g* for 15 min. The pellet was rinsed and resuspended in 50 mM Tris–HCl (pH 8.0) with 0.1% Triton X-100 and 1 mg/mL lysozyme in a ratio of 1 g of WCW per 10 mL. A 30 mL resuspended cell mixture was subjected to sonication (37.5 W, 3s on/5s off, 45% amplitude, for 10 min) and centrifuged at 12,000*g* for 15 min to isolate the soluble and insoluble fractions. The pellet was rinsed with a 0.5% SDS solution in 50 mM Tris–HCl (pH 8.0) until no discernible bacterial pellet remained, after which the subsequent purification processes were similar to those used in CFPS-expressed spidroin.

### 4.6 Quantification of Spidroin and mEGFP

Spidroins purified from a 200 mL batch culture were lyophilized and resuspended in Milli-Q water and then aliquoted into two equal vials: one was centrifuged and resuspended in 1 mL of CFPS-mimicking solution (30% cell extract, 25% 4× master mix), while the other was lyophilized and weighed. The resuspended sample in the CFPS-mimicking solution was diluted into various samples: 1, 2, 4, 10, 20, 40, 100, 200, and 1000× dilutions. Simple regression analysis was conducted to analyze the relationship between the observed RFU and the estimated concentration (dry weight in milligrams multiplied by the dilution ratio per milliliter). The RFU of R_2_8-G and R_1_8-R was measured at their corresponding wavelengths (mEGFP: Ex 485/Em 525 nm and mCherry: Ex 587/Em 610 nm). Regression equations are provided in Figures S4 and 10 for R_2_8-G and R_1_8-R, respectively.

For mEGFP quantification, mEGFP was overexpressed in BLR(DE3) Δ*endA* and purified through a His tag and nickel resin column (described in detail in Method S2). The protein concentration was assessed using the Bradford assay and analyzed in relation to the measured RFU (the regression equation is provided in Figure S2).

### 4.7 SDS-PAGE and Western Blotting of Spidroins

Spidroin samples for SDS-PAGE and Western blotting analyses were prepared according to the following procedures: spidroin samples were solubilized in HFIP, diluted CFPS sample to 30 μL by 50 mM Tris–HCl (pH 8.0), combined with 10 μL of 4× LDS loading dye (EBL Biotechnology), and heated at 100 °C for 10 min. Post-treated spidroin samples were subsequently put onto a 10% polyacrylamide gel, which was run at 110 V for 1.5–2 h and then stained with Coomassie Brilliant Blue R-250.

For Western blotting, spidroins prerun in SDS-PAGE were gently placed on the PVDF membrane and transferred by an electrical blotter (Mini Trans-Blot, Bio-Rad) at 400 mA for 90 min. The membranes were subsequently incubated at 4 °C in gelatin-NET buffer overnight for blocking. The next day, an anti-His tag primary antibody (rabbit origin; Bioman Scientific) at a dilution ratio of 1:2500 in gelatin-NET buffer was used to specifically target the His-tag on the spidroin products for 1 h. Membranes were washed three times with PBST solution before applying the secondary antibody (5000-fold dilution of goat antirabbit IgG in gelatin-NET buffer; PerKinElmer) preconjugated with HRP. Following a 1 h incubation at 37 °C, excess antibodies were removed by washing with PBST three times. The membrane was subjected to the Western Lightning ECL reagent (Revvity) and examined using an imaging system (UVP BioSpectrum 500; Analytik Jena).

### 4.8 Microscopy, LLPS, and Fiber Spinning/Observation

Purified spidroin and nonpurified CFPS reaction mixtures were examined under a fluorescence microscope. In the LLPS and spidroin spinning tests, purified spidroin was solubilized in HFIP, and 2 μL of the solution was injected into a 10 μL NH_4_Pi bath droplet preplaced on a transparent glass slide. A microtip was immersed in the solution mixture and gradually drawn with a shear force to facilitate potential fiber production. Under appropriate circumstances, fiber would manifest; otherwise, LLPS or dissolution in the buffer would occur. A coverslip was then promptly affixed to the glass slide and examined under the microscope.

### 4.9 Data Analysis

Linear regression was performed with Microsoft Excel. Other statistics and plotting were performed by the R script.

### 4.10 Material Information

All Chemicals used in this research are listed in Table S3.

## Supporting Information Available

DNA sequences, plasmid map, and additional experimental details and methods (PDF)

## Abbreviations

CFPS: cell-free protein synthesis
MaSp: major ampullate spidroin
NTD: N-terminal domain
NDCR: charge-reversed
CTD: C-terminal domain
R_2_8-G: NTD-R_2_8-CTD-mEGFP
R_1_8-R: NTD-R_1_8-CTD-mCherry
CMC: critical micelle concentration
NH_4_Pi: ammonium phosphate
DLS: dynamic light scattering
SDS: sodium dodecyl sulfate
HFIP: hexafluoro-iso-propanol
