# Supplementary material for: Developing an E. coli-Based Cell-Free Protein Synthesis System for Artificial Spidroin Production and Characterization
Source: ACS Synth Biol. 2025 Apr 21;14(5):1829–42. doi: 10.1021/acssynbio.5c00241 (PMC12090345; doi:10.1021/acssynbio.5c00241)
Supplement: Supplementary file 1 — sb5c00241_si_001.pdf [file sb5c00241_si_001.pdf]

Supplemental Information

Developing an *E. coli*-Based Cell-Free Protein Synthesis System for Artificial Spidroin Production and Characterization

Chang-Yen Huang[1], Ruei-Chi Wang[1], Tzy-Shyuan Hsu[1], Tzu-Ning Hung[1], Ming-Yan Shen[1], Chung-Heng Chang[1], and Hsuan-Chen Wu[1*]

Affiliation
[1]Department of Biochemical Science and Technology, National Taiwan University, 106319 Roosevelt Road, Da-an District, Taipei City, Taiwan, Republic of China.

[*]Corresponding author:
Hsuan-Chen Wu
Department of Biochemical Science and Technology, National Taiwan University
No. 1, Section 4, Roosevelt Road, Taipei 10617, Taiwan (ROC)
Tel: +886-2-3366-4525
Fax: +886-2-2363-4796
Email: hcwu7@ntu.edu.tw

Supporting Information

Supplemental Figures S1-16
Supplemental Methods S1-2
Supplemental Tables S1-3

Supplemental Figures

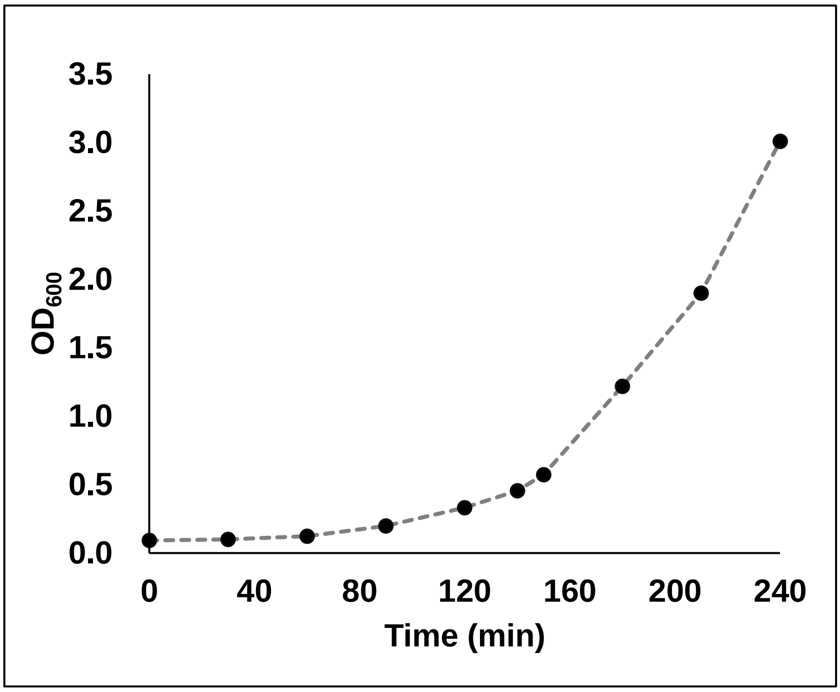

Supplemental Figure S1. Growth curve of *E. coli* BLR(DE3) *ΔendA* and T7 RNA polymerase induction. The initial inoculation density was at OD_{600} = 0.01. 1 mM IPTG was added once the OD_{600} reached 0.5 (t = 140 min) and cells were harvested for CFPS cell extract preparation once OD_{600} reached 3.0 (t = 240 min).

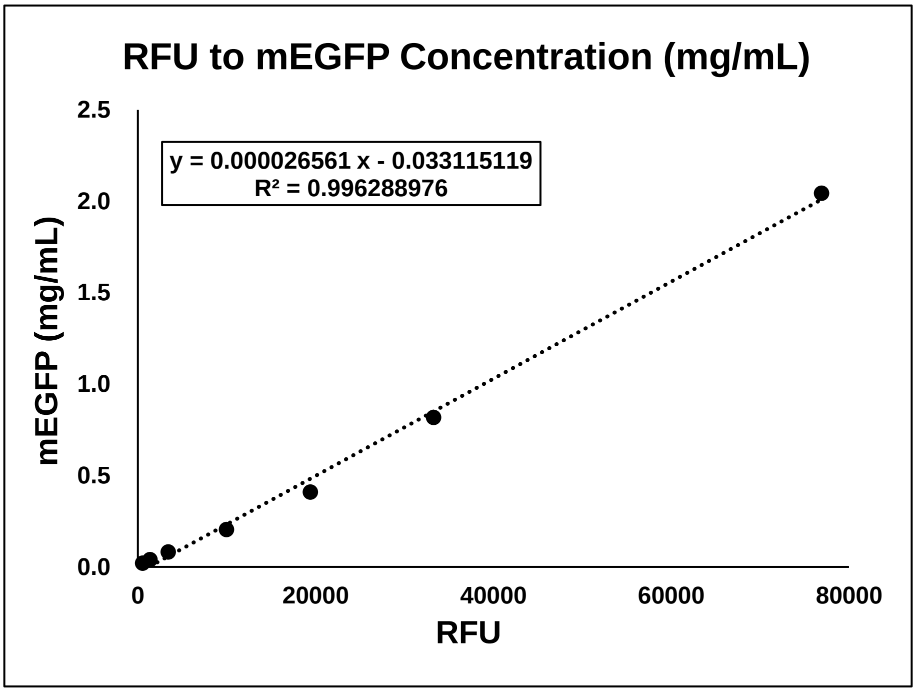

Supplemental Figure S2. Regression analysis of mEGFP relative fluorescence units (RFU) vs concentration (mg/mL). Purified mEGFP was measured with the Bradford assay, followed by a serial dilution. Each dilution was assessed for its RFU and then regressed on the concentration.

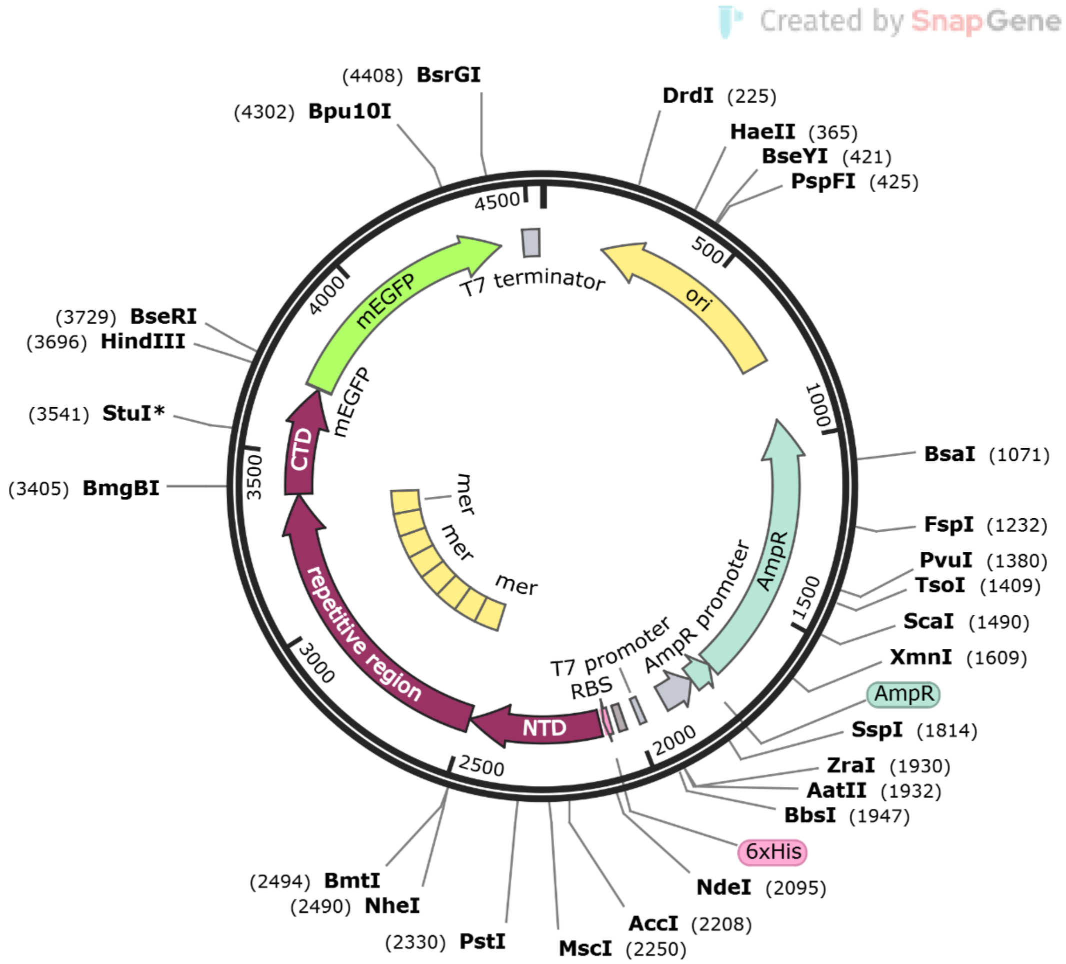

Supplemental Figure S3. Plasmid map of pCFPS-NTD-R$_2$8-CTD-mEGFP (pCFPS-R$_2$8-G).

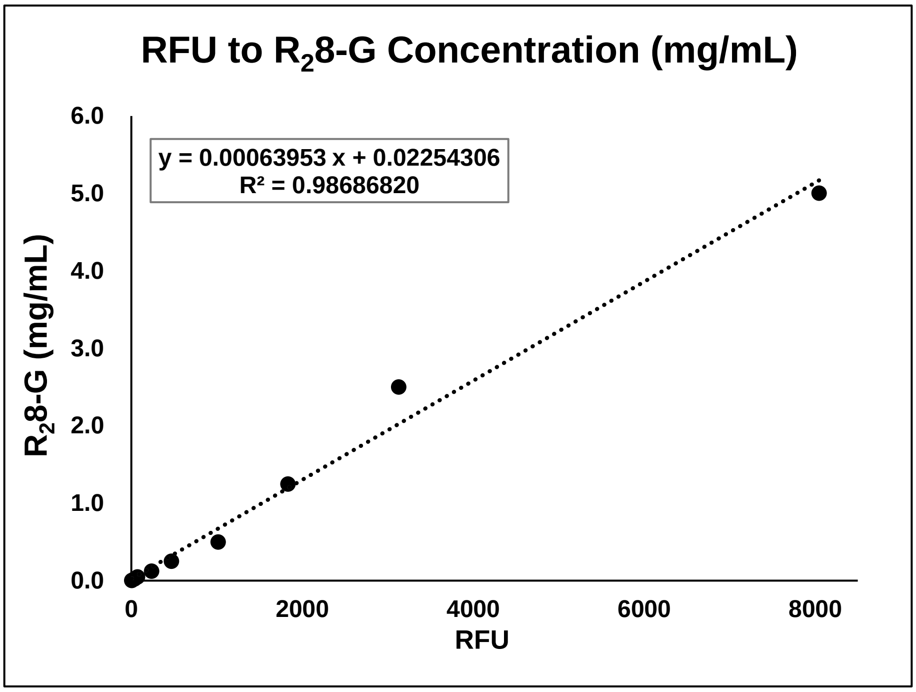

Supplemental Figure S4. Correlation between $R_2$8-G RFU and its concentration (mg/mL). *In vivo*-produced $R_2$8-G was purified and lyophilized. Subsequently, the dried pellet was re-suspended in CFPS solution (using cell extract as the control) and subjected to serial dilution (1, 2, 4, 10, 20, 40, 100, 200, 1000X dilution) for RFU measurement. Each dilution was assessed for its RFU and then regressed on the concentration.

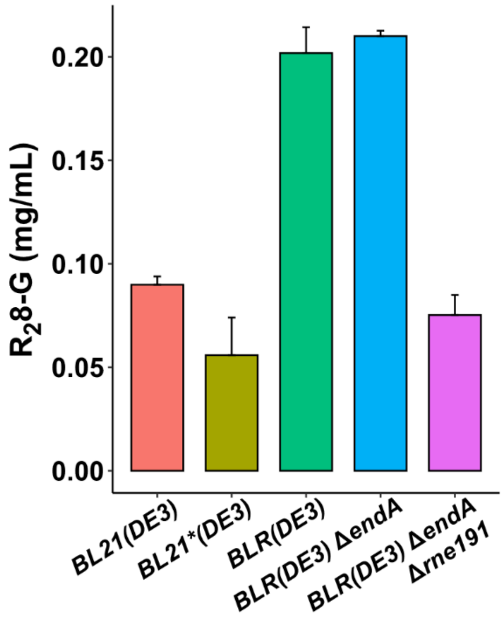

Supplemental Figure S5. Spidroin yield comparison among five DE3-strains, which showed a similar trend as observed in mEGFP synthesis. Noted that BLR(DE3) *ΔendA* gave a highest yield in average, as compared to BLR(DE3).

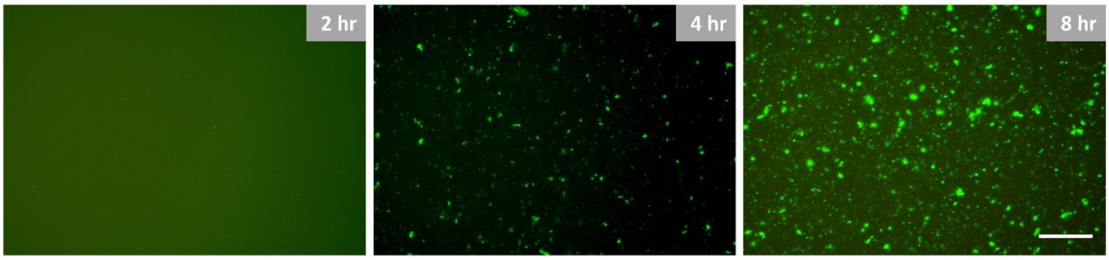

Supplemental Figure S6. Time-course microscopic observation of $R_2$8-G in the CFPS reaction mixture at t = 2, 4, and 8 hr. The insoluble green particles appeared after 4 and 8 hr, but not after 2 hr. Scale bar = 50 μm.

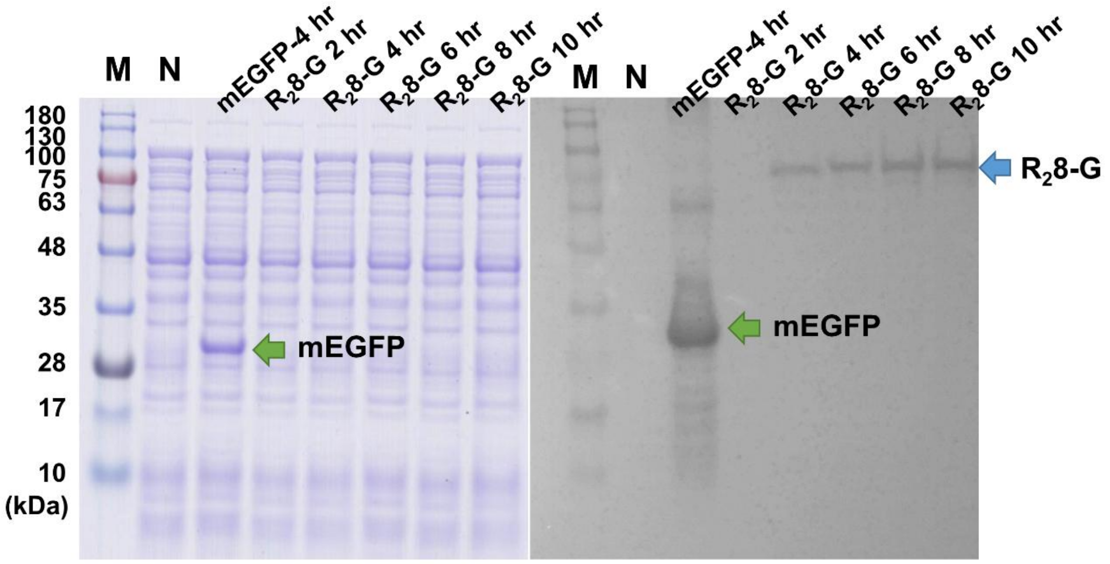

Supplemental Figure S7. SDS-PAGE and Western blotting of $R_2$8-G via CFPS reaction (2-10 hr) and mEGFP (4 hr). M: protein marker. N: no-plasmid CFPS control group at t = 12 hr. mEGFP and $R_2$8-G products are indicated by the green and blue arrows, respectively.

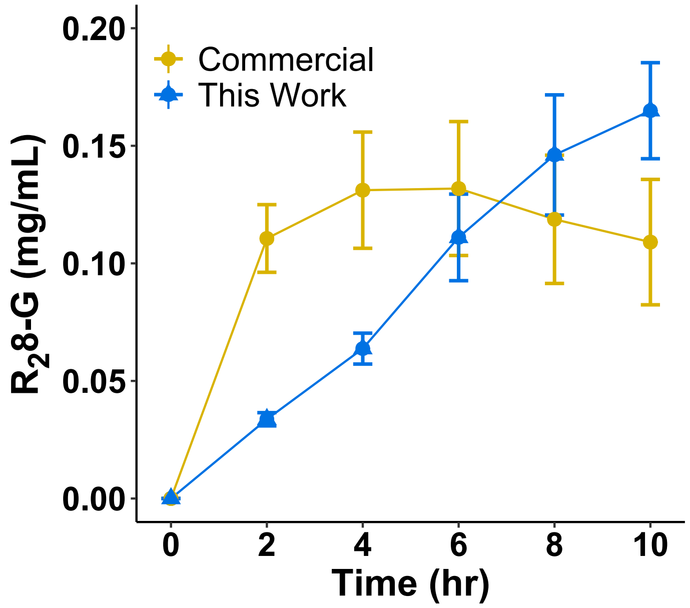

Supplemental Figure S8. Yield comparison of $R_2$8-G using RFU signals between the commercial CFPS kit (NEBExpress®, New England Biolabs) and the CFPS system employed in this research. The RFU reading from the commercial kit reached a plateau at t = 4 hr while our CFPS system matched it at t = 8 hr and exceeded it at t = 10 hr. CFPS experiments were conducted in triplicate.

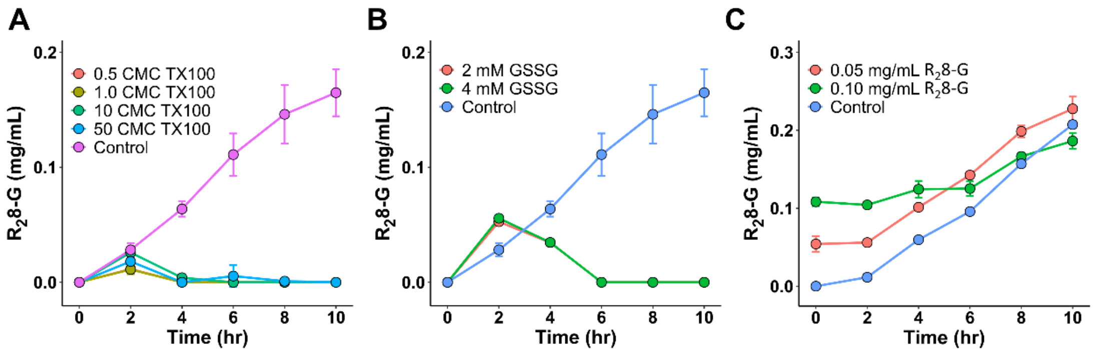

Supplemental Figure S9. Optimization of CFPS with the supplementation of Triton X-100, GSSG, and pre-prepared $R_2$8-G to enhance the production of $R_2$8-G. (A-C) Various amounts of Triton X-100, GSSG, and pre-made $R_2$8-G were included respectively, although no notable enhancement in $R_2$8-G synthesis was detected. Experiments were performed in triplicate.

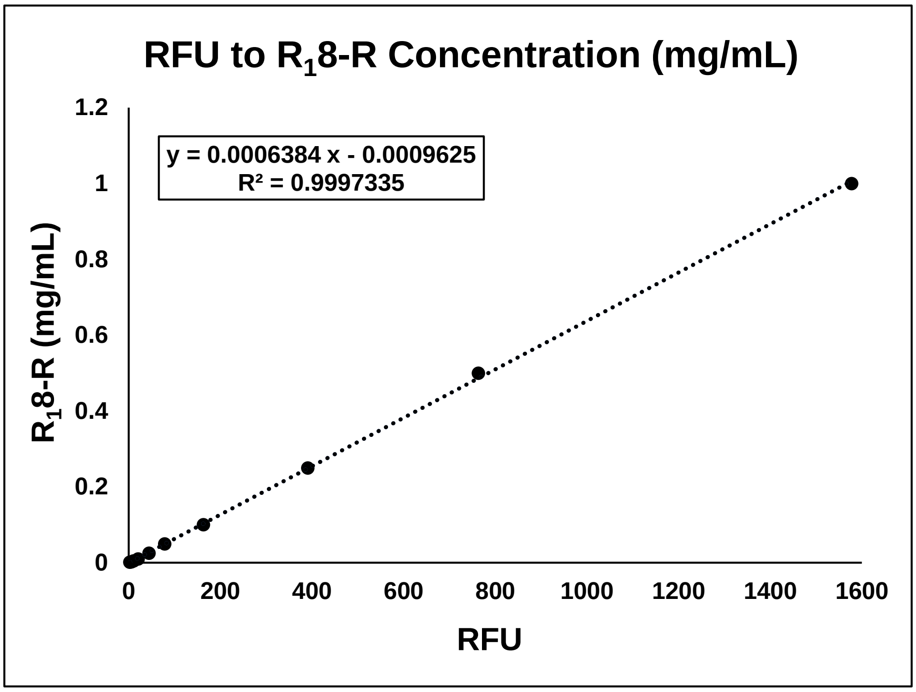

Supplemental Figure S10. Correlation between $R_1$8-R RFU and its concentration. *In vivo*-produced $R_1$8-R was purified and lyophilized. Afterward, the dried pellet was re-suspended in CFPS solution (using cell extract as the control) and subjected to serial dilution (1, 2, 4, 10, 20, 40, 100, 200, 1000X dilution) for RFU measurement. Measured RFU within the linear range was assessed and regressed on the concentration (mg/mL).

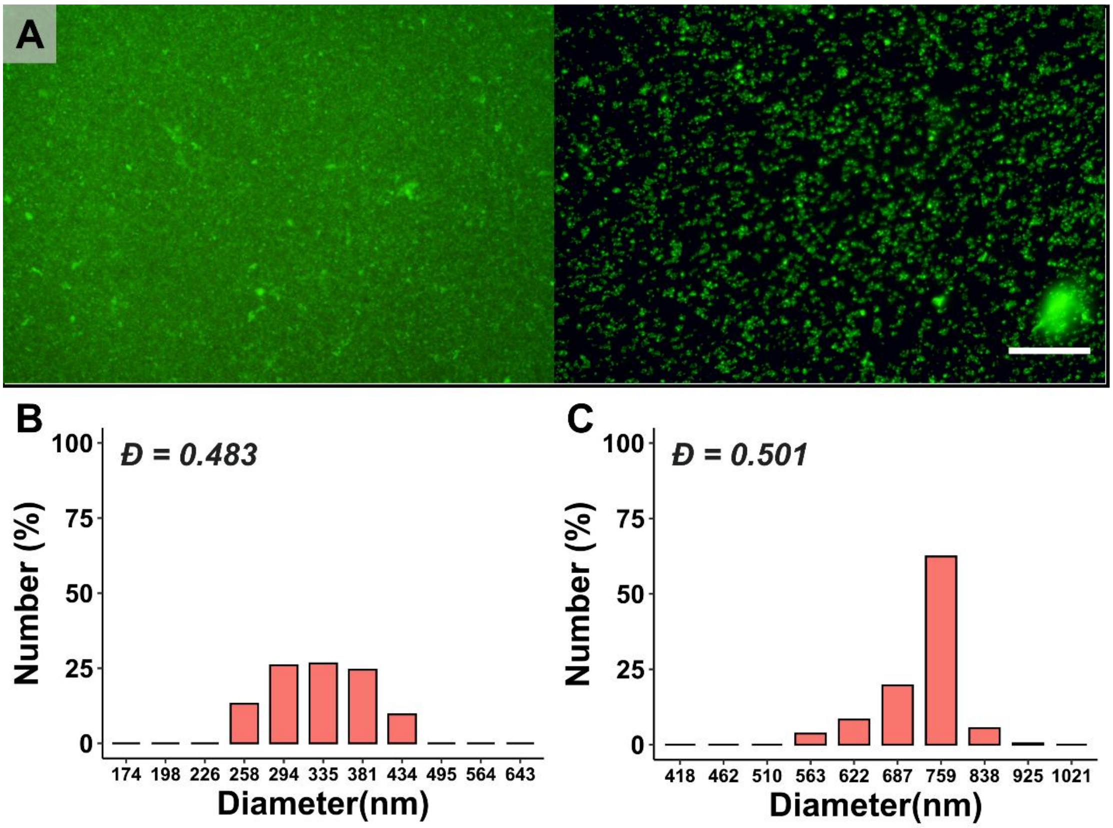

Supplemental Figure S11. Size measurement of R_28-G. (A) Purified R_28-G was re-suspended in Milli-Q water (left) and NH_4Pi buffer pH 8.0 (right). Upon resuspension in a low ionic environment (water), the R_28-G samples exhibited reduced size and increased background fluorescence, suggesting micelle-like particles' dissociation. Scale bar = 50 µm. (B, C) DLS analysis of R_28-G in Milli-Q water (B) and NH_4Pi (C) environment, with the polydispersity index (PDI, *Đ*) shown in the plot respectively. The size difference can be correlated to the microscopic observation.

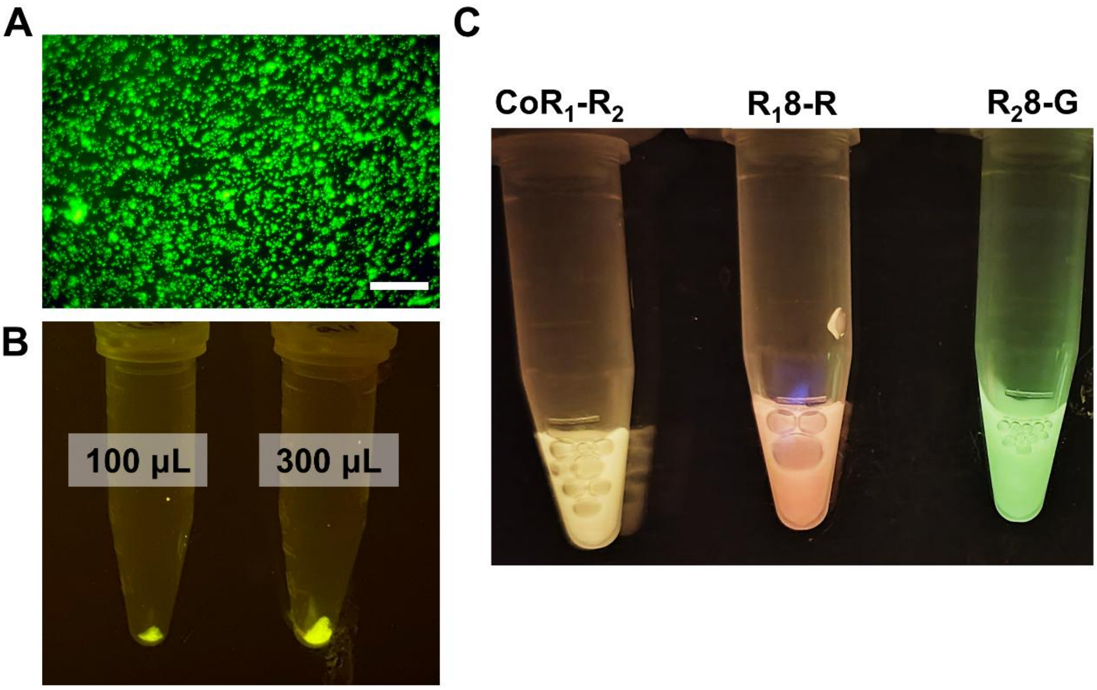

Supplemental Figure S12. (A) Examination of NDCR-$R_2$8-G products in the CFPS reaction via fluorescence microscopy. Scale bar = 50 µm. (B) Observation of purified $R_2$8-G pellets from 100 µL (left) and 300 µL (right) of the CFPS reaction under the fluorescent light. (C) CFPS solution of CoR_1-R_2, $R_1$8-R, and $R_2$8-G after 10-hr CFPS reaction under the excitation of a fluorescent light.

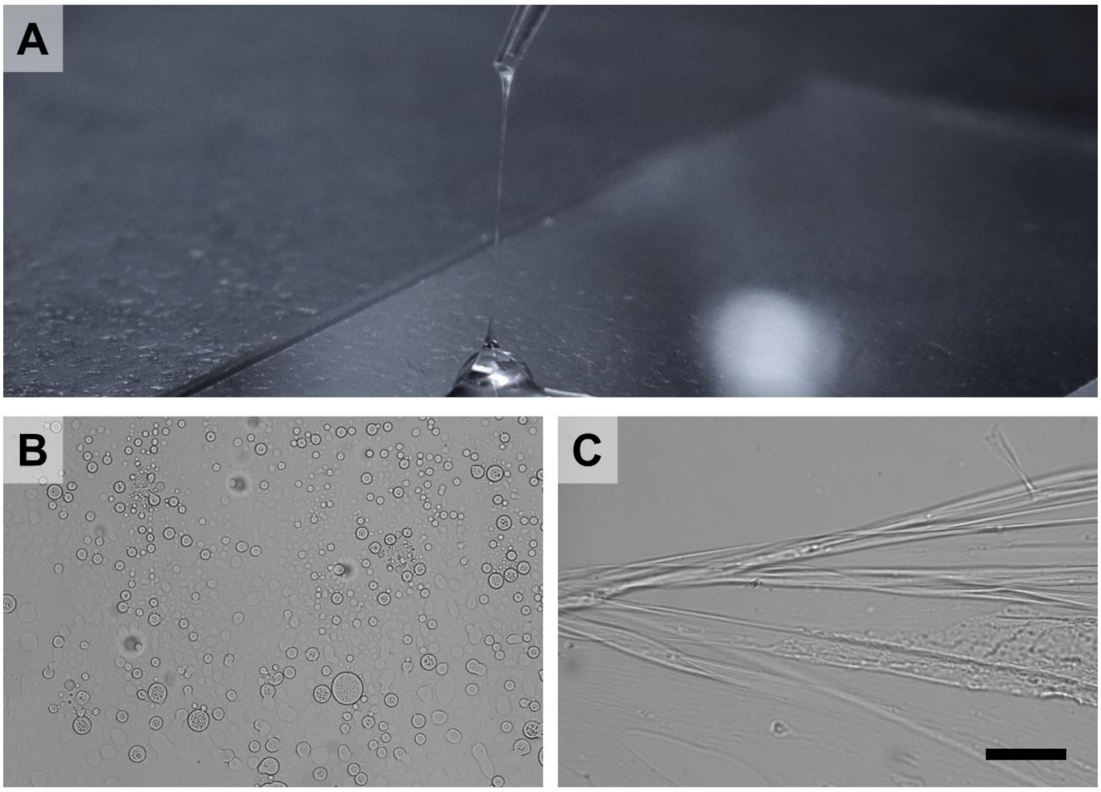

Supplemental Figure S13. Fiber formation with the CFPS-synthesized spidroin. (A) $R_2$8-G fiber spinning using microtip dragging in NH_4Pi bath at pH 4.3. (B and C) At pH 8.0, NDCR-$R_2$8-G exhibited LLPS, but at pH 4.3, it displayed a scattered fibril-type pattern. Scale bar = 50 μm.

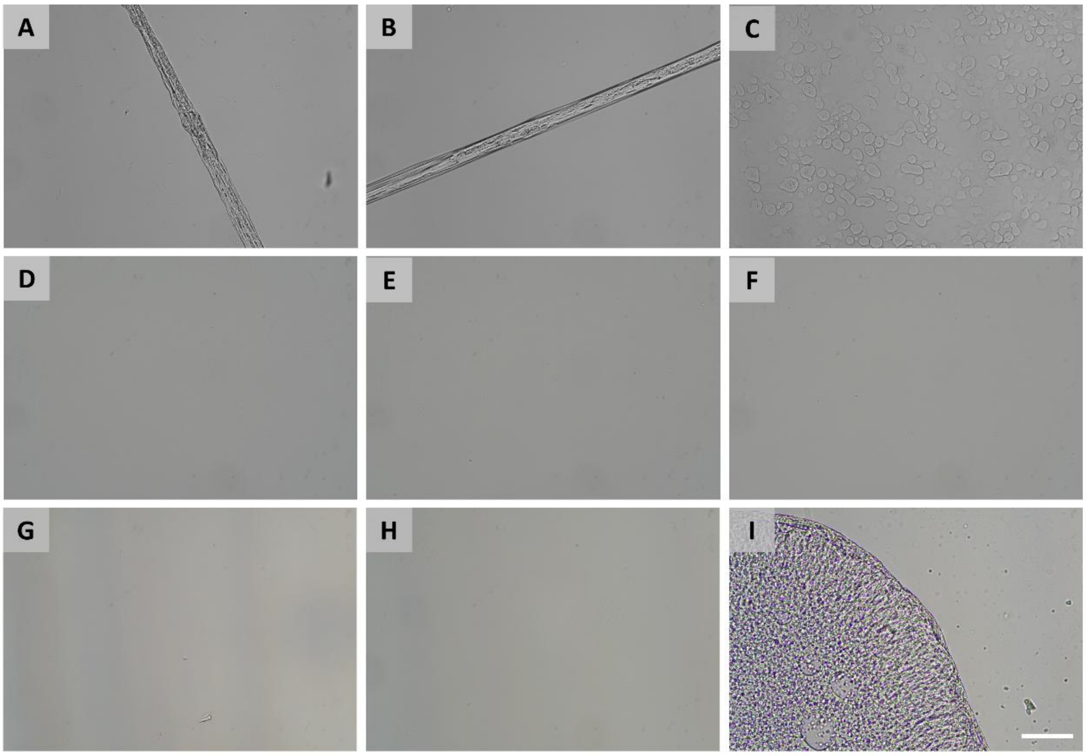

Supplemental Figure S14. (A-C) $R_2$8-G, $R_1$8-R, and NDCR-$R_2$8-G were introduced into a pH 2.8 NH_4Pi solution for the spinning test. (D-F) No significant structure was observed after the injection of $R_2$8-G, $R_1$8-R, and NDCR-$R_2$8-G into Milli-Q water, resulting only in a homogeneous solution. (G-I) BSA dissolved in HFIP (5% wt/vol) was introduced into Milli-Q water and NH_4Pi bath at basic and acidic pH levels (8.0 and 2.8), respectively. Scale bar = 50 µm.

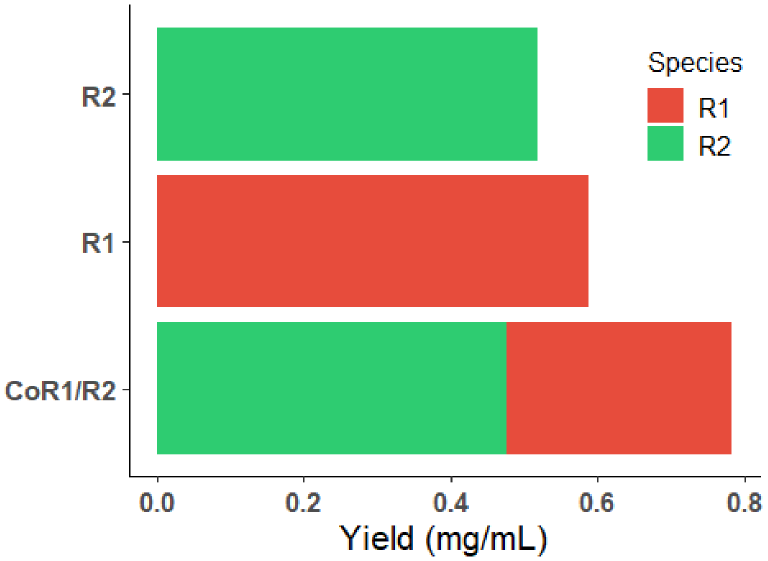

Supplemental Figure S15. The yield of $R_1$8-R and $R_2$8-G in the CoR_1/R_2 group, transformed from the RFU reading.

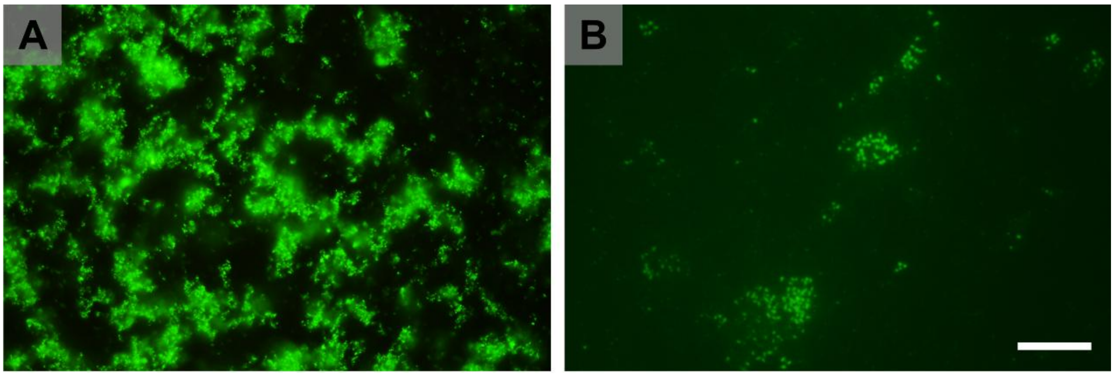

Supplemental Figure S16. CFPS production of NDCR-$R_1$32/$R_1$128-CTD-mEGFP. (A) Synthesized $R_1$32-G and (B) $R_1$128-G (non-purified) were examined under a fluorescence microscope, which existed as insoluble micelle-like particles. Scale bar = 20 μm.

Supplemental Methods

Supplemental Method S1: **Production and purification of T7 RNA polymerase.** Plasmid pT7RP was transformed into BLR(DE3) *ΔendA* competent cells through heat-shock transformation for overexpression. Single colonies were picked and inoculated into 5 mL LB medium with 25 μg/mL chloramphenicol in the culture tube. The culture was incubated at 37°C, 250 rpm overnight. Overnight culture was then inoculated into fresh LB medium (200 mL in flask, 25 μg/mL chloramphenicol) at 1% ratio (OD_{600} ~ 0.01) and cultured at 37°C, 250 rpm until the OD_{600} reached 0.5. 1 mM IPTG was further supplemented and the condition was switched to 25°C, 250 rpm followed by 16-18 hr incubation. The culture was harvested at 4°C, 8000 g centrifugation for 15 minutes, followed by multiple rinsing with a loading buffer (10 mM Tris-HCl, 100 mM NaCl, 5 mM β-mercaptoethanol, 5% glycerol, pH 8.0). Cells were re-suspended with the loading buffer in the concentration of 1 g WCW/10 mL, followed by probe sonication (37.5 W, 3s on/5s off, 45% amplitude, for 10 minutes) to obtain the lysed extract. The lysate was clarified through centrifugation at 4°C, 8000 g for 15 minutes and a filtration on the supernatant using 0.20 μm syringe filters (Sartorius®). T7RP was further purified by Ni-NTA column (Ni Sepharose™ High Performance, Cytiva) as per the supplier's recommendation[1]. Purified T7RP was concentrated to 5-10 mg/mL via 50 kDa Amicon Ultra Centrifugal tube (Merck) and stored at -20 °C with the buffer substituted with a storage buffer (40 mM K_2HPO_4, 2 mM DTT, 2 mM EDTA, pH 8.0). T7RP was quantified through the Bradford assay.

Supplemental Method S2: Production, purification, and quantification of mEGFP. Plasmid pET28a-mEGFP was transformed into BLR(DE3) *ΔendA* competent cell via heat shock for overexpression. The overexpression was conducted similarly as the case of T7RP, except the antibiotic was switched to 50 μg/mL kanamycin and the condition after IPTG-induction was 30°C, 250 rpm for 8 hr. Cells were then harvested and stored at -20°C for no more than one week. Upon purification, the cell pellet was rinsed with Tris buffer (50 mM Tris-HCl, pH 8.0) and lysed via probe sonication. The downstream purification process was similar to T7RP's instead the buffer was replaced with the Tris buffer, and purified mEGFP was not followed by ultracentrifugation and concentrating. Purified mEGFP was quantified through the Bradford assay. mEGFP was further aliquoted into different dilution ratios, and their RFU at Ex 485/Em 520 nm was measured. The RFU was regressed with the corresponding concentration determined (mg/mL).

Supplemental Tables

Supplemental Table S1. DNA and the amino acid sequences of NTD, NDCR, CTD, R_1, $R_2{}^2$, mEGFP, and mCherry.

**Name**	**DNA sequence**	**Amino acid sequence**
NTD	ATGCAAAACACCCCATGGTCAAGCA<br>CGGCACTGGCAGATGCATTCATCAA<br>TGCATTTTTGAACGAAGCTGGAAGA<br>ACTGGAGCTTTTACTGCGGATCAAC<br>TCGATGACATGTCTACCATTGGTGAC<br>ACTCTCAAAGGAGCAATGGACAAA<br>ATGGCCAGAAGTAACAAGAGTTCCA<br>AATCGAAGTTGCAAGCTCTTAACAT<br>GGCATTTGCATCATCAATGGCGGAA<br>ATTGCTGCAGTGGAGCAAGGAGGC<br>ATGGGTGTTGAAGCGAAAACAAAC<br>GCTATTGCGGATTCCTTAAACGCAG<br>CATTCATGCAGACCACTGGATCAATA<br>AATAGTCAGTTTGTGAATGAAATCA<br>GAAGTTTGATTAGCATGTTTGCACA<br>AGCGTCAGCAAATGAAGTA	MQNTPWSSTALADAFINAFLNE<br>AGRTGAFTADQLDDMSTIGDTL<br>KGAMDKMARSNKSSKSKLQAL<br>NMAFASSMAEIAAVEQGGMGV<br>EAKTNAIADSLNAAFMQTTGSI<br>NSQFVNEIRSLISMFAQASANEV
NDCR	ATGCAAAACACCCCATGGTCAAGCA<br>CGGCACTGGCAGATGCATTCATCAA<br>TGCATTTTTGAACGAAGCTGGAAGA<br>ACTGGAGCTTTTACTGCGGATCAAC<br>TCGATAAAATGTCTACCATTGGTGAC<br>ACTCTCAAAGGAGCAATGGACAAA<br>ATGGCCAGAAGTAACAAGAGTTCCA<br>AATCGGACTTGCAAGCTCTTAACAT<br>GGCATTTGCATCATCAATGGCGGAA<br>ATTGCTGCAGTGGAGCAAGGAGGC<br>ATGGGTGTTGAAGCGAAAACAAAC<br>GCTATTGCGGATTCCTTAAACGCAG<br>CATTCATGCAGACCACTGGATCAATA<br>AATAGTCAGTTTGTGAATGAAATCA<br>GAAGTTTGATTAGCATGTTTGCACA<br>AGCGTCAGCAAATGAAGTA	MQNTPWSSTALADAFINAFLNE<br>AGRTGAFTADQLDKMSTIGDTL<br>KGAMDKMARSNKSSKSDLQAL<br>NMAFASSMAEIAAVEQGGMGV<br>EAKTNAIADSLNAAFMQTTGSI<br>NSQFVNEIRSLISMFAQASANEV
R_1	GGCGCTGGTGCAGCGGCTGCCGCA	GAGAAAAAASGAGQGGYGRQ

	GCGTCTGGTGCGGGTCAGGGTGGCT ACGGACGTCAAGGCGGTCAAACTA GT	GGQTS
R_2	GGCCCTGGAGGCTACGGTCCAGGCC AACAGGGCCCATCTGGGCCGGGTTC TGCGGCTGCGGCAGCCGCTGCGGC AGGACCGGGAGGTTACGGTCCAGG ACAGCAAACTAGC	GPGGYGPGQQGPSGPGSAAAA AAAAGPGGYGPGQQTS
CTD	GGAGCCTCTGCCGCTGCATCACGTC TCTCTTCTCCTGAAGCTAGTTCTAGA GTTTCTTCTGCCGTTTCTAATTTGGT GTCAAGTGGCCCAACCAATTCCGCT GCTTTGTCGAATACTATTAGTAATGT GGTATCTCAAATTAGCTCAAGTAAC CCAGGCCTTTCAGGATGCGATGTTC TTGTTCAAGCCCTTTTGGAAGTCGT TTCAGCTCTTATTCATATCTTAGGATC GTCTAGCATTGGTCAAGTAAATTATG GTTCTGCTGGACAGGCTACTCAAAT CGTTGGTCAATCTGTGTATCAAGCCC TTGGTAAGCTT	GASAAASRLSSPEASSRVSSAVS NLVSSGPTNSAALSNTISNVVSQI SSSNPGLSGCDVLVQALLEVVS ALIHILGSSSIGQVNYGSAGQAT QIVGQSVYQALG
mEGFP	GTGAGCAAGGGCGAGGAGCTGTTC ACCGGGGTGGTGCCCATCCTGGTCG AGCTGGACGGCGACGTAAACGGCC ACAAGTTCAGCGTGTCCGGCGAGG GCGAGGGCGATGCCACCTACGGCAA GCTGACCCTGAAGTTCATCTGCACC ACCGGCAAGCTGCCCGTGCCCTGGC CCACCCTCGTGACCACCCTGACCTA CGGCGTGCAGTGCTTCAGCCGCTAC CCCGACCACATGAAGCAGCACGACT TCTTCAAGTCCGCCATGCCCGAAGG CTACGTCCAGGAGCGCACCATCTTC TTCAAGGACGACGGCAACTACAAG ACCCGCGCCGAGGTGAAGTTCGAG GGCGACACCCTGGTGAACCGCATCG AGCTGAAGGGCATCGACTTCAAGG AGGACGGCAACATCCTGGGGCACA	VSKGEELFTGVVPILVELDGDVN GHKFSVSGEGEGDATYGKLTLK FICTTGKLPVPWPTLVTTLTYGV QCFSRYPDHMKQHDFFKSAMPE GYVQERTIFFKDDGNYKTRAEV KFEGDTLVNRIELKGIDFKEDGN ILGHKLEYNYNSHNVYIMADKQ KNGIKVNFKIRHNIEDGSVQLAD HYQQNTPIGDGPVLLPDNHYLS TQSKLSKDPNEKRDHMVLLEFV TAAGITLGMDELYK

	AGCTGGAGTACAACTACAACAGCCA<br>CAACGTCTATATCATGGCCGACAAG<br>CAGAAGAACGGCATCAAGGTGAAC<br>TTCAAGATCCGCCACAACATCGAGG<br>ACGGCAGCGTGCAGCTCGCCGACC<br>ACTACCAGCAGAACACCCCCATCGG<br>CGACGGCCCCGTGCTGCTGCCCGAC<br>AACCACTACCTGAGCACCCAGTCCA<br>AGCTGAGCAAAGACCCCAACGAGA<br>AGCGCGATCACATGGTCCTGCTGGA<br>GTTCGTGACCGCCGCCGGGATCACT<br>CTCGGCATGGACGAGCTGTACAAG	
mCherry	GTGAGCAAGGGCGAGGAGGATAAC<br>ATGGCGATCATCAAAGAGTTCATGC<br>GCTTCAAAGTCCACATGGAAGGCAG<br>CGTTAATGGTCACGAGTTCGAAATT<br>GAGGGCGAAGGCGAAGGTCGTCCG<br>TATGAGGGTACACAGACCGCTAAAC<br>TGAAAGTCACGAAAGGTGGTCCAC<br>TGCCATTTGCTTGGGATATTCTGAGC<br>CCACAGTTCATGTATGGCTCCAAAG<br>CCTATGTGAAACATCCGGCCGATATT<br>CCGGACTATCTGAAACTGAGCTTCC<br>CTGAAGGGTTCAAATGGGAACGTGT<br>GATGAACTTTGAGGATGGTGGTGTT<br>GTGACAGTGACACAGGATTCTAGCC<br>TGCAAGACGGTGAGTTCATCTATAA<br>AGTGAAACTGCGTGGCACGAATTTT<br>CCGAGTGATGGCCCGGTTATGCAGA<br>AAAAAACGATGGGTTGGGAGGCCT<br>CTAGTGAGCGTATGTATCCAGAAGA<br>TGGCGCTCTGAAAGGCGAAATCAA<br>ACAGCGTCTGAAACTGAAAGATGGT<br>GGCCACTATGATGCCGAAGTGAAAA<br>CCACGTATAAAGCCAAAAAACCTGT<br>CCAACTGCCTGGTGCCTATAACGTT<br>AACATCAAACTGGACATCACCTCAC<br>ACAATGAGGACTATACGATCGTGGA	VSKGEEDNMAIIKEFMRFKVHM<br>EGSVNGHEFEIEGEGEGRPYEGT<br>QTAKLKVTKGGPLPFAWDILSPQ<br>FMYGSKAYVKHPADIPDYLKLS<br>FPEGFKWERVMNFEDGGVVTV<br>TQDSSLQDGEFIYKVKLRGTNFP<br>SDGPVMQKKTMGWEASSERMY<br>PEDGALKGEIKQRLKLKDGGHY<br>DAEVKTTYKAKKPVQLPGAYN<br>VNIKLDITSHNEDYTIVEQYERA<br>EGRHSTGGMDELYK

	GCAGTATGAGCGTGCTGAAGGACGT CATTCTACCGGCGGCATGGACGAGC TGTACAAG	

Supplemental Table S2. Detailed information on plasmids and strains used in the research.

**Plasmid/Strain**	**Description**
pCFPS-mEGFP	T7 promoter-driven monomeric enhanced-green fluorescence protein (mEGFP) expression plasmid designed for CFPS expression. Amp^R.
pCFPS-mCherry	T7 promoter-driven monomeric red fluorescence protein (mCherry) expression plasmid designed for CFPS expression. Amp^R.
pCFPS-$R_2$8-G	T7 promoter-driven 8-repeated R2 spidroin (NTD and CTD-mEGFP flanked) expression plasmid designed for CFPS expression. Amp^R.
pCFPS-$R_1$8-R	T7 promoter-driven 8-repeated R1 spidroin (NTD and CTD-mCherry flanked) expression plasmid designed for CFPS expression. Amp^R.
pET28a-mEGFP	T7 promoter-driven, *lacO*-controlled mEGFP expression plasmid. Kam^R
pT7RP	T7 promoter-driven, *lacO*-controlled T7 RNA polymerase expression plasmid for T7RP overexpression and purification. Cm^R.
BL21(DE3)	*Escherichia coli* B Strain *fhuA2 [lon] ompT gal (λ DE3) [dcm] ΔhsdS.* T7 expression strain with OmpT and Lon proteases deficiency for better protein production.
BL21*(DE3)	BL21(DE3) Δ*rne131. rne131* deficiency allows better mRNA retention in its cytosol.
BLR(DE3)	BL21(DE3) Δ*recA. recA* deficiency prevents potential recombination within DNAs.
BLR(DE3) Δ*endA*	*endA* deficiency allows better plasmid retention in its cytosol.
BLR(DE3) Δ*endA* Δ*rne131*	

Supplemental Table S3. Chemicals and media used in this research

**Name**	**Brand**	**Product No.**
Ammonium phosphate, monobasic	Sigma-Aldrich	216003

AMP (Adenosine 5' monophosphate)	Sigma-Aldrich	A2754-1G
Ampicillin sodium	Cyrusbioscience	101-69-52-3
ATP (Adenosine 5' triphosphate)	Sigma-Aldrich	A1852
β-mercaptoethanol	Sigma-Aldrich	M6250
BSA (Bovine serum albumin)	Sigma-Aldrich	05470-5G
Chloramphenicol	Cyrusbioscience	101-56-75-7
CMP (Cytidine 5' monophosphate)	Sigma-Aldrich	C1006-1G
CBR-R250 (Coomassie brilliant blue R250)	Bio-Basic	CB0037
DTT (Dithiothreitol)	Cyrusbioscience	101-3483-12-3
D-Glucose	Bioshop	GLU501.1
EDTA-2Na	Sigma-Aldrich	SI-E4844-500G
Gelatin	Sigma-Aldrich	48723-500G
Glutathiol	Sigma-Aldrich	G4376-1G
Glycerol	J.T.Baker	2136-08
Glycine	Bio-basic	GB0235
GMP (Guanosine 5' monophosphate)	Sigma-Aldrich	G8377-1G
GSSG (Glutathiol, L-glutathione oxidized)	Sigma-Aldrich	G4376-1G
HFIP (Hexafluoro-iso-propanol)	Thermo Scientific	A12747.22
IPTG (Isopropyl β-D-1-thiogalactopyranoside)	Cyrusbioscience	101-367-93-1
Kanamycin sulfate	Cyrusbioscience	101-25389-94-0
LB broth	Cyrusbioscience	21100
L-Alanine	Sigma-Aldrich	A7627-100G
L-Arginine		A8094-25G
L-Asparagine		A0884-25G
L-Aspartic acid		A9256-100G
L-Cysteine		C7352-25G
L-Glutamic acid		G8415-100G
L-Glutamine		G8540-25G
L-Histidine		H8000-10G
L-Isoleucine		I2752-5G
L-Leucine		L8000-25G

L-Lysine		L5501-5G
L-Methionine		M9625-5G
L-Phenylalanine		P2126-100G
L-Proline		P5607-25G
L-Serine		S4500-100G
L-Threonine		T8625-10G
L-Tryptophan		T0254-1G
L-Tyrosine		T8566-25G
L-Valine		V0500-25G
Magnesium acetate, tetrahydrate	Scharlau	P201405030057
Magnesium glutamate, tetrahydrate	Sigma-Aldrich	49605-250G
PEG-8000	Sigma-Aldrich	1546605
Potassium acetate	Sigma-Aldrich	236497-100G
Potassium glutamate, monohydrate	Sigma-Aldrich	G1501-100G
Potassium oxalate, monohydrate	Sigma-Aldrich	P0963-100G
Potassium phosphate dibasic	J.T.Baker	3251-2.5KG
Potassium phosphate monobasic	Fluka	60220-1KG
SDS (Sodium dodecyl sulfate)	NEOFROXX	250KG001
Sodium chloride	Sigma-Aldrich	S9888-1KG
Spermidine	Sigma-Aldrich	SI-S2626-1G
Tris-acetate	Sigma-Aldrich	T1258
Tris base	Bio-Basic	TB0196
Triton X-100	Bio-Basic	TB0198
Tryptone	Bio-Basic	TG217
Tween 20	J.T.Baker	X251-07
UMP (Uridine 5' monophosphate)	Sigma-Aldrich	U6375-1G
Yeast extract	Bio-Basic	G0961

Supplemental Reference

[1] Merck. (n.d.) Purification of Histidine-Tagged Recombinant Proteins Using Ni Sepharose® High Performance.

[2] Chen, W.-C., Wang, R.-C., Yu, S.-K., Chen, J.-L., Kao, Y.-H., Wang, T.-Y., Chang, P.-Y., Sheu, H.-S., Chen, S.-C., Liu, W.-R., Yang, T.-I., and Wu, H.-C. (2023) Self-Healable Spider Dragline Silk Materials, *Advanced Functional Materials 33*, 2303571.
